# From omics to AI—mapping the pathogenic pathways in type 2 diabetes

**DOI:** 10.1002/1873-3468.70115

**Published:** 2025-07-17

**Authors:** Siobhán O'Sullivan, Lu Qi, Pierre Zalloua

**Affiliations:** ^1^ Department of Biological Sciences College of Medicine and Health Sciences, Khalifa University Abu Dhabi UAE; ^2^ Tulane University Obesity Research Center, School of Medicine Tulane University New Orleans LA USA; ^3^ Department of Epidemiology and Public Health College of Medicine and Health Sciences, Khalifa University Abu Dhabi UAE; ^4^ Harvard T.H. Chan School of Public Health Boston MA USA

**Keywords:** artificial intelligence, clinical translation, digital twins, multi‐omics integration, precision medicine, systems biology, type 2 diabetes

## Abstract

Understanding the biochemical pathways and interorgan cross talk underlying type 2 diabetes (T2D) is essential for elucidating its pathophysiology. These pathways provide a mechanistic framework linking molecular dysfunction to clinical phenotypes, enabling patient stratification based on dominant metabolic disturbances. Advances in multi‐omics, including genomics, transcriptomics, proteomics, microbiomics, and metabolomics, offer a systems‐level view connecting genetic variants and regulatory elements to disease traits. Single‐cell technologies further refine this perspective by identifying cell‐type‐specific drivers of β‐cell failure, hepatic glucose dysregulation, and adipose inflammation. AI‐driven analytics and machine learning integrate these high‐dimensional datasets, uncovering molecular signatures and regulatory networks involved in insulin signaling, lipid metabolism, mitochondrial function, and immune‐metabolic cross talk. This review synthesizes current evidence on T2D's molecular architecture, emphasizing key pathways such as PI3K‐Akt, AMPK, mTOR, JNK, and sirtuins. It also explores the role of gut microbiota in modulating host metabolism and inflammation. Adopting a pathway‐centric systems biology approach moves beyond statistical associations toward mechanistic insight. Integrating multi‐omics with AI‐based modeling represents a transformative strategy for stratifying patients and guiding precision therapies in diabetes care.

**Impact statement**

This review translates complex biochemical pathways into therapeutic direction for type 2 diabetes, addressing a critical gap between molecular research and clinical care. By integrating multi‐omics, AI, and systems biology, it empowers the scientific community to develop targeted interventions that reduce the global burden of this escalating metabolic disease.

## Graphical abstract

Integrating multi‐omics data with AI‐based modelling (unsupervised and supervised machine learning) identify optimal patient clusters, informing AI‐driven accurate risk stratification. Digital twins simulate individual trajectories in real time, guiding precision medicine by matching patients to targeted therapies. Continuous longitudinal monitoring tracks shifts in molecular endophenotypes over time, enabling adaptive treatment adjustments and improving outcomes in T2D.

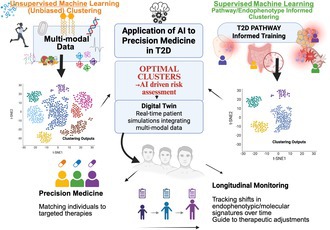

## Abbreviations

**AI**, artificial intelligence

**Akt**, protein kinase B

**AMPK**, AMP‐activated protein kinase

**ATP**, adenosine triphosphate

**BCAA**, branched chain amino acids

**CDKN2A**, cyclin‐dependent kinase inhibitor 2A

**CGM**, continuous glucose monitor

**CRP**, C‐reactive protein

**CVD**, cardiovascular disease

**DT**, digital twin

**eIF4E**, eukaryotic initiation factor 4E

**ER**, endoplasmic reticulum

**ERAD**, ER‐associated degradation

**ETC**, electron transport chain

**FFAs**, free fatty acids

**GFAT**, Glutamine:Fructose‐6‐Phosphate Amidotransferase

**GLP**, glucagon‐like peptide

**GLP‐1 RA**, glucagon‐like peptide 1 receptor agonist

**GLP‐RA**, glucagon‐like peptide receptor agonist

**GLUT4**, glucose transporter type 4

**GPX**, glutathione peroxidase

**GSH**, reduced glutathione

**GWAS**, genome‐wide association studies

**HbA1c**, hemoglobin A1c

**HBP**, hexosamine biosynthetic pathway

**HDL**, high‐density lipoprotein

**HNF1B**, hepatocyte nuclear factor 1 beta

**IFNγ**, interferon gamma

**IL‐1β**, interleukin 1 beta

**IL‐6**, interleukin 6

**IR**, insulin resistance

**IRS1**, insulin receptor substrate 1

**JNK**, Jun N‐terminal kinase

**LDL**, low‐density lipoprotein

**LKB1**, liver kinase B1

**LPS**, lipopolysaccharide

**MAPK**, mitogen‐activated protein kinase

**MARD**, mild age‐related diabetes

**MENA**, middle east, North Africa

**ML**, machine learning

**MOD**, mild obesity‐related diabetes

**MSEA**, marker set enrichment analysis

**mTOR**, mechanistic target of rapamycin

**mTORC1**, mechanistic target of rapamycin complex 1

**NF‐κB**, nuclear factor kappa‐light‐chain‐enhancer of activated B cells

**NGS**, next‐generation sequencing

**NIT‐1**, cultured pancreatic β‐cells

**NLRP3**, NOD‐, LRR‐ and pyrin domain‐containing protein 3

**NO**, nitric oxide

**NOD**, non‐obese diabetic

**NOS**, nitric oxide synthase

**NOX**, NADPH oxidase

**PDX1**, pancreatic and duodenal homeobox 1

**PGC‐1α**, peroxisome proliferator‐activated receptor gamma coactivator 1‐alpha

**PI3K**, phosphoinositide 3‐kinase

**PP2A**, protein phosphatase 2A

**PPAR**, peroxisome proliferator‐activated receptor

**PRS**, polygenic risk score

**PTEN**, phosphatase and tensin homolog

**RNN**, recurrent neural networks

**ROS**, reactive oxygen species

**SCFA**, short‐chain fatty acids

**SERCA**, sarco‐endoplasmic reticulum ATPases

**ShAP**, shapley additive explanations

**SIDD**, severe insulin‐dependent diabetes

**SIRD**, severe insulin‐resistant diabetes

**SIRT**, sirtuin family

**SLC16A11**, solute carrier family 16 member 11

**SNP**, single‐nucleotide polymorphism

**SOD**, superoxide dismutase

**SREBP**, sterol regulatory element‐binding protein

**STAT**, signal transducer and activator of transcription

**SVM**, support vector machine

**T2D**, type 2 diabetes

**TBC1D4**, TBC1 domain family member 4

**TGF‐β**, transforming growth factor beta

**TLR**, toll‐like receptor

**TNF‐α**, tumor necrosis factor alpha

**TRX**, thioredoxin

**UCP**, uncoupling protein

**UDP‐GlcNAc**, uridine diphosphate N‐acetylglucosamine

**VAT**, visceral adipose tissue

**WGS**, whole‐genome sequencing

**XAI**, explainable artificial intelligence

**XGBoost**, extreme gradient boosting

Approximately 463 million adults aged 20–79 years have *diabetes mellitus*, mainly type 2 diabetes (T2D), a figure projected to rise to 700 million by 2045, classifying it as a global pandemic [1]. T2D is characterized by tissue‐specific insulin resistance (IR) and pancreatic β‐cell dysfunction, driven by an interplay of local tissue abnormalities and systemic disruptions in interorgan cross talk. The failure of β‐cells to compensate for IR disrupts glucose and lipid homeostasis, underlying the metabolic disturbances central to T2D pathogenesis.

T2D arises from a combination of environmental, metabolic, and behavioral determinants, as well as the composition and richness of the gut microbiota, along with numerous genetic variants influencing key biochemical pathways. The generalized hormonal effect of insulin is on multiple tissues including the pancreas, liver, adipose tissue, muscle, and intestine, further emphasizing the systemic nature of the disease. Chronic hyperglycemia in T2D is linked to inflammatory responses and altered metabolites, highlighting the inflammatory component of its pathology.

Understanding the biochemical pathways (and interorgan cross talk) central to T2D is essential for dissecting its pathophysiology and identifying targets for prevention, diagnosis, and treatment [2]. Recent advances in multi‐omics technologies (genomics, transcriptomics, proteomics, microbiomics, metabolomics) provide a systems‐level perspective, linking genetic variants and regulatory elements to specific phenotypes. Single‐cell analyses further enhance this understanding by uncovering cell‐type‐specific contributions to β‐cell dysfunction, hepatic glucose dysregulation, and adipose inflammation.

Unraveling the complexity of T2D can certainly benefit from computational approaches, such as machine learning (ML) and artificial intelligence (AI), to analyze high‐dimensional, multi‐layered data generated by multi‐omics studies. ML excels at identifying nonlinear associations and hidden patterns across genetic, transcriptomic, and metabolomic layers, while AI‐powered models predict disease risk, progression, and therapeutic responses with increasing precision. High‐throughput computing facilitates integration of these insights, enabling the identification of regulatory networks and pathways critical to T2D.

Different patients exhibit different metabolic dysfunctions owing to the heterogeneity of this complex disease. Translational research thus gains from a pathway‐centric approach because clinical interventions often target specific steps or regulators within these pathways. Investigating the metabolic pathways that underpin T2D ensures that cutting‐edge technologies (high‐throughput and integrative multi‐omics platforms coupled with bioinformatics and computational pathway analysis tools such as KEGG, Reactome, and Gene Ontology) remain connected to the underlying biology of T2D. Pathways provide a lens to dissect this complex heterogeneity, providing an integrative framework that connects molecular insights into disease mechanisms, clinical phenotypes, and therapeutic interventions. This allows for the stratification of patients based on their dominant metabolic disturbances and lays the groundwork for precision medicine in T2D.

This review explores several metabolic and signaling pathways disrupted in T2D, as well as the interorgan cross talk that governs metabolic homeostasis, and how it becomes dysregulated in disease states [2, 3, 4]. It also explores the potential impact of integrating these metabolic and signaling pathways using multi‐omics technologies, single‐cell analyses, and AI‐driven computational tools to investigate the complex pathophysiology of T2D. By aligning these molecular layers, key metabolic dysfunctions that drive patient heterogeneity can be uncovered. Advancing this systems‐level understanding of these molecular drivers is essential to bridge the gap between basic biological research and clinical translation.

## Epidemiology of T2D

Approximately 90% of diabetic patients have T2D [5] while the remaining 10% have type I diabetes and other rarer forms [6]. The incidence of T2D increases with age [5] peaking on average at 55–59 years, and presenting in men slightly earlier than in women. T2D now ranks seventh among the leading causes of disability and years of life lost (DALYs) [5, 7]. Globally, the incidence and prevalence of T2D continue to rise. Current estimates indicate that every 6 s, a person dies from diabetes or its complications, with about half of these deaths occurring in individuals under 60 years old [8, 9]. This distribution pattern aligns with socio‐economic development and lifestyle changes [10, 11]. These have led to declines in nutritional quality and increases in sedentary behavior driving increased overweight and obesity in regions that were previously predominantly burdened by undernutrition [11, 12]. The COVID‐19 pandemic—through lockdowns that further increased sedentary behavior and worsened food insecurity—has further accelerated these trends, contributing to higher T2D incidence [13, 14]. The rise has been most dramatic in countries undergoing epidemiological transitions, particularly in Asia, the Middle East, and North Africa (MENA) [15, 16].

## The complex genetic architecture of T2D

T2D, like many complex disorders, is a non‐stochastic, nonlinear, epigenetically polygenic model [17]. Its risk factors cluster into identifiable biological patterns rather than occurring by chance. Genetic predisposition, modulated by epigenetic modifications, intersects with environmental factors, creating a multidimensional network of dysregulated metabolic pathways. The dynamic progression of T2D is driven by complex feedback loops, cross talk between key metabolic tissues (adipose, liver, muscle, and pancreas), and modifiable environmental factors (e.g., diet, physical activity). These interactions culminate in progressive insulin resistance (IR) and β‐cell dysfunction, collectively driving T2D pathogenesis [17, 18, 19].

**Fig. 1:**
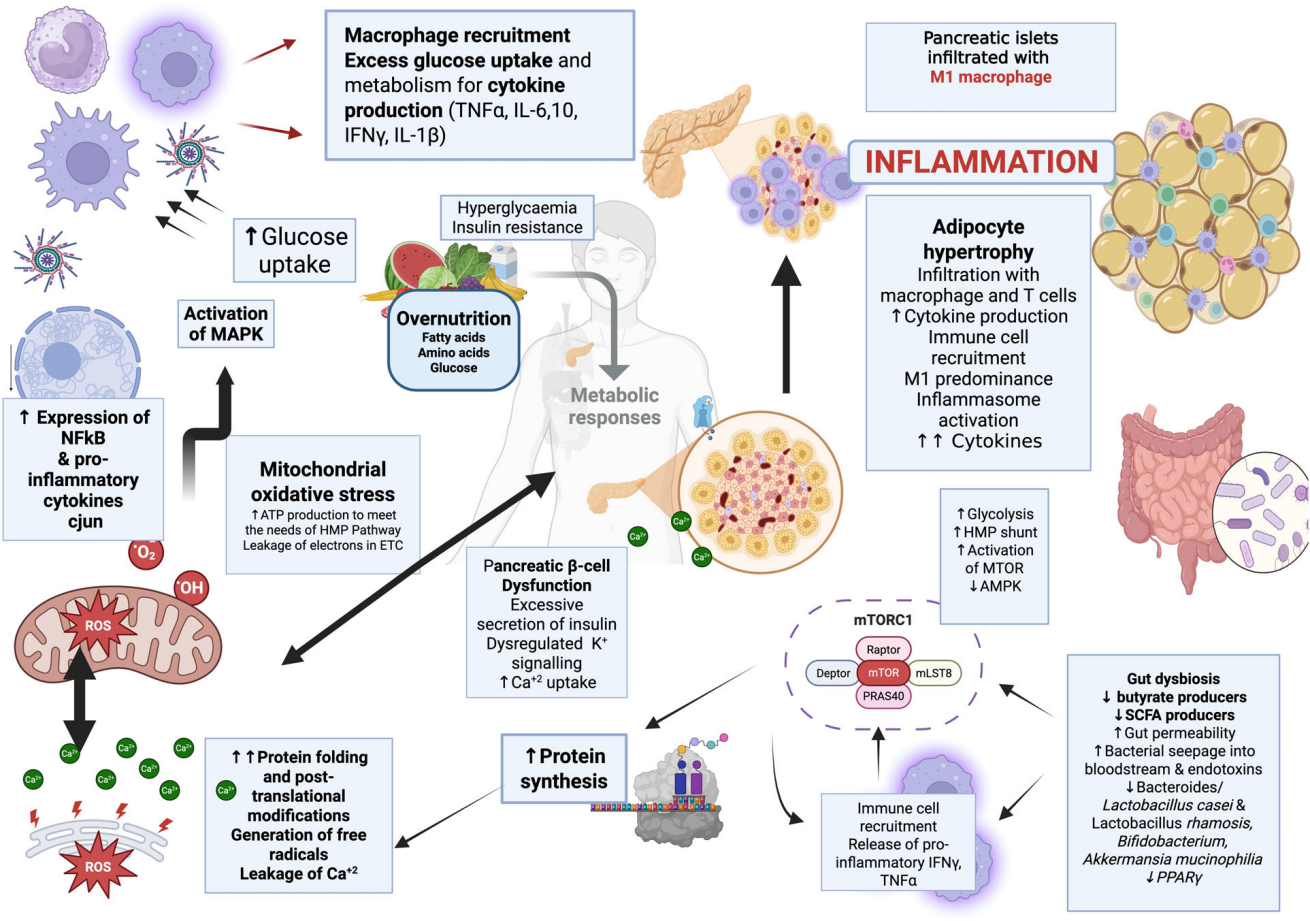
Metabolic signatures of T2D; interconnected metabolic, inflammatory, and cellular pathways driving T2D pathophysiology. Overnutrition, driven by excess intake of fatty acids, amino acids, and glucose, triggers hyperglycemia and insulin resistance, contributing to β‐cell dysfunction. Pancreatic β‐cell stress presents as excessive insulin secretion, dysregulated potassium signaling, and increased calcium uptake, amplifying metabolic disturbances. Mitochondrial oxidative stress plays a central role, with increased ATP production to meet metabolic demands leading to electron leakage in the ETC. This results in the generation of ROS, which drive protein misfolding, calcium leakage, and further oxidative damage, exacerbating β‐cell dysfunction. Activation of the MAPK pathway promotes the expression of NF‐κB and pro‐inflammatory cytokines, transcription factors e.g., c‐Jun, macrophage recruitment, and immune cell infiltration in pancreatic islets and adipose tissue. Inflammatory M1 macrophages accumulate, producing cytokines (e.g., TNF‐α, IL‐6, IFNγ, and IL‐1β), reinforcing systemic inflammation. Adipocyte hypertrophy and immune cell infiltration promote further cytokine production, resulting in inflammasome activation and worsening IR. Gut dysbiosis, characterized by reduced populations of butyrate and short‐chain fatty acid SCFA producers, increases gut permeability and endotoxin leakage into the bloodstream, further driving inflammation. Beneficial microbial populations, including *Lactobacillus casei*, *Lactobacillus rhamnosus*, *Bifidobacterium*, and *Akkermansia muciniphila*, are diminished, contributing to metabolic dysfunction through downregulation of *PPARγ*. The mTORC1 pathway, activated by nutrient overload, enhances glycolysis and the HMP shunt while suppressing AMPK signaling. This promotes immune cell recruitment and the release of pro‐inflammatory cytokines, perpetuating metabolic and cellular stress. Created in BioRender.

At the heart of T2D etiology lies a network of interconnected molecular and metabolic pathways that govern β‐cell function, insulin sensitivity, and systemic glucose regulation. While T2D is diagnosed on the basis of a single metabolite (glucose), hyperglycemia can arise due to multiple complex etiological processes that vary between individuals [20, 21]. Overnutrition, in the context of excess caloric intake and nutrient overload, plays a pivotal role in initiating and exacerbating these processes. It promotes tissue‐specific IR and pancreatic β‐cell dysfunction by contributing to lipotoxicity, glucotoxicity, and chronic low‐grade inflammation. These pathological mechanisms arise from the interplay of local abnormalities within various tissues and dysregulation of tissue cross talk, ultimately driving T2D pathogenesis (Fig. 1).

## Integrative multi‐omics and AI‐driven systems biology: Addressing functional bottlenecks in T2D

The clinical outcome variability in treatment response and disease evolution routinely observed with T2D has prompted many investigations into the underlying molecular heterogeneity. Multi‐omics technologies spanning genomics, transcriptomics, proteomics, and metabolomics have served as powerful tools providing some insights into the etiology and progression of T2D pathophysiology. The etiology of T2D is driven by a strong hereditary predisposition contributing an estimated 20–80% of disease susceptibility, while environmental exposures throughout life modulate the remaining risk [19, 22]. Genome‐wide association studies (GWAS) marked a revolutionary shift in understanding T2D genetics, moving from hypothesis‐driven research on candidate genes to a discovery‐driven unbiased survey of the entire genome [23]. GWAS has identified numerous biochemical pathways and potential mechanisms of disease, offering invaluable insights into complex genetic interactions [24]. Variants in genes such as *TCF7L2*, *HHEX*, *KCNJ11*, *CENTD2*, *HNF1B*, *CDKAL1*, *SLC30A8*, CDKN2A, 2B, *IRS1*, *PPARα*, and *IGF2BP2* are shown to be strongly associated with susceptibility to T2D and related phenotypes across populations [25, 26, 27, 28, 29, 30, 31, 32, 33, 34, 35, 36]. Notably, many of these variants map to genes involved in β‐cell function, insulin secretion, and the activity of insulin‐sensitive tissues (liver, adipose tissue, muscle). However, some associated loci have no direct association with insulin production or glucose metabolism, leaving their function in disease pathogenesis ambiguous. Overcoming this functional bottleneck is essential for moving from gene discovery to understanding the complex pathophysiology of T2D.

Ultimately, while GWAS represented a methodological leap, it fell short of fulfilling its initial promise of deciphering the full genetic architecture of T2D and was not the panacea to the “missing heritability” problem. Most genetic variants identified in GWAS confer only modest risk, collectively explaining about 20% of T2D's heritability [37]. Linking GWAS associations to specific causal variants or high‐impact variants remains challenging. GWAS primarily capture the additive effects of individual single‐nucleotide polymorphisms (SNPs), overlooking the interplay of multiple genes and gene–environment interactions. The assumption that the combined effect of genetic variants is additive does not hold true for most complex diseases. Moreover, many association signals lie in non‐coding regions of the genome that are difficult to interpret biologically. Translation of GWAS findings into clinical practice has been fraught with challenges. While GWAS uncovered numerous genetic markers, few have led to actionable medical interventions or have been incorporated into routine clinical care. This translational gap reflects the complexity of moving from statistical associations to validated clinical applications. Meanwhile, GWAS inherently failed to capture a substantial portion of the genetic landscape (e.g., rare variants, structural variants, epigenetic mechanisms, or gene–gene interactions) contributing to disease susceptibility. To address this, international consortia, such as GoT2D and T2D‐GENES, employed advanced genomic technologies (whole‐genome sequencing and targeted sequencing) to discover rare and low‐frequency variants that were not detected by earlier GWAS studies. Notable examples include variants in genes like *SGSM2*, *MADD*, *TBC1D30*, *KNK1*, *PAM*, *CCNDS2*, *PDX1*, *MTNR1B*, *HNF1*, and *G6PC2*, illustrating the utility of sequencing technologies in uncovering functional variants beyond common SNPs [38].

Epigenetic modifications further complicate T2D's genetic landscape. For example, aberrant histone acetylation in pancreatic islets can induce β‐cell death or inactivate glucose transporters in muscle, causing hyperinsulinemia and hyperglycemia [19, 39]. Beyond acetylation, the chromatin landscape is shaped by various other epigenetic modifications (methylation, phosphorylation, ubiquination, SUMOylation) each contributing uniquely to gene regulation [40]. Non‐coding RNAs (ncRNAs) and the epitranscriptome represent another facet of epigenetics. Many ncRNAs are transcribed but not translated, and they can orchestrate epigenetic modifications that silence or activate gene expression [41].

Array‐based DNA methylation profiling and whole‐genome sequencing have been used to map methylation changes at single‐nucleotide resolution, revealing the functional impact of altered methylation on transcription [42]. Large numbers of T2D‐associated DNA methylated regions are in the same regions as T2D candidate genes identified by GWAS, such as *TCF7L2*, *ADCY5*, *KCNQ1*, and *GLIS3*. These show both genetic associations and diabetes‐related methylation changes. Functional studies in β‐cells confirm the importance of some of these genes (e.g., *TCF7L2* and *ADCY5*) in insulin secretion and glucose homeostasis [19].

Tools such as expression quantitative trait loci (eQTL) mapping have further enhanced functional genomics by linking specific variants to changes in gene expression, providing insights into the biological mechanisms by which risk alleles drive T2D. Combining *cis*‐ and *trans*‐eQTL mapping with GWAS results has been instrumental in identifying T2D signatures and advancing our understanding of disease heterogeneity [43, 44]. Next‐generation sequencing (NGS) based transcriptional profiling techniques have enabled the study of miRNA‐mediated transcriptional responses in individual cells and cell types [45, 46]. Transcriptome analyses have been used to identify upregulated and downregulated genes in T2D. High‐throughput transcriptome sequencing has identified differential gene expression in tissues relevant to T2D, which helps explain variations in glucose metabolism, IR, and insulin secretion. Such differential gene expression patterns impact β‐cell function and survival and highlight genes involved in pro‐inflammatory pathways and other metabolic disturbances in T2D.

Despite the many advances brought by large‐scale sequencing research, eQTL mapping, and other genomics‐related tools, the results have been contrary to expectations. Rare and low‐frequency variants play a less significant role in understanding the complex architecture of T2D than initially anticipated [37]. While such variants may have larger effects individually, they represent only a modest portion of the overall genetic risk. They also fail to replicate across populations and frequently occur in non‐coding regions, making it challenging to infer their biological function in T2D pathophysiology [47, 48]. Low initial sample sizes were a limitation due to insufficient power to identify the associations (large sample sizes are required to detect rare‐variant associations due to their low frequency). Even with large consortia such as the UK Biobank, detecting and validating the effects of rare variants remains limited [49]. These insights marked another disappointing chapter in the ongoing struggle to understand the complexity of the disease and its genetic architecture [50, 51].

This ‘vertical’ approach of examining the cascade from genotype through to phenotype demands a thorough interrogation of the biological mechanisms at play, as it is by mapping these trajectories that we gain insights into gene expression dynamics, protein synthesis, and metabolic shifts that drive T2D progression. Each omics layer offers complementary unique insights: genomics captures inherited susceptibility, epigenetics reflects gene–environment interactions, while transcriptomics, proteomics, and especially metabolomics are dynamic reflections of short‐term metabolic perturbations, inflammatory shifts and treatment effects (Table 1).

**Table 1:** Functional roles and representative biomarkers across omics layers in T2D.

Omics layer	Functional relevance	Examples of biomarkers
Genomics	Assesses inherited genetic susceptibility to T2D, identifying risk loci involved in insulin secretion and β‐cell function	SNPs in TCF7L2, SLC30A8, PPARγ
Epigenomics	Captures environmental regulation of gene expression via DNA methylation and histone modifications	DNA methylation in TCF7L2, histone modifications in metabolic tissues
Transcriptomics	Quantifies gene expression changes associated with inflammation, insulin resistance, and β‐cell dysfunction	↑ IL1β, ↑ TNF‐α in immune cells; ↓ insulin transcripts in β‐cells
Proteomics	Profiles tissue‐specific proteins reflecting metabolic signaling and insulin action	↑ CRP, ↑ leptin, ↓ adiponectin, ↓ GLUT4 in insulin‐resistant states
Metabolomics	Measures small molecules indicating altered metabolic flux and oxidative stress	↑ BCAAs, ↑ ceramides, ↑ lactate
Lipidomics	Detects lipid species involved in lipotoxicity and inflammation	↑ Ceramides (e.g., d18:1/18:0), ↑ S1P in adipose tissue
Microbiomics	Analyzes gut microbial composition and function in regulating metabolism and inflammation	↑ LPS, ↑ *Firmicutes/Bacteroidetes* ratio, ↓ *Akkermansia muciniphila*
Single‐cell omics	Dissects cell‐type‐specific molecular profiles in tissues affected by T2D	scRNA‐seq of islets showing β‐cell dedifferentiation and immune infiltration
Spatial omics	Maps localized expression of immune and metabolic signals during disease progression	IL1β and TNF‐α near β‐cells infiltrated by macrophages

Single‐cell omics technologies (e.g., scRNA‐seq and spatial transcriptomics) provide an even more precise picture of T2D at the cellular level, revealing cell‐type‐specific metabolic rewiring, particularly in early disease stages. Such tools can identify early molecular changes or biomarkers in vulnerable cell types, including pancreatic β‐cells, adipocytes, hepatocytes, or immune cells that are otherwise hidden in bulk tissue data. For example, profiling adipose‐resident macrophages distinguishes pro‐inflammatory M1 from anti‐inflammatory M2 states, clarifying the immune–metabolic axis dysregulation in T2D disease progression. Integration analyses of epigenomic and transcriptomic data have helped in identifying which variants are active in pancreatic β‐cells, hepatocytes, or adipocytes, the main tissues affected in T2D, enabling individual cell‐targeted therapies and guiding subtype‐specific interventions, for example, targeting lipotoxicity or inflammation‐driven T2D subtypes [52].

Systems biology provides a lens to interpret multi‐omics data. Instead of viewing hundreds of T2D‐associated loci in isolation, systems approaches organize loci into pathways, highlighting the common pathways and the factors that drive disease phenotypes. Mapping these variants to regulatory networks helps assign variants to T2D‐specific pathways. For instance, eQTL analyses map non‐coding variants to gene expression profiles (eQTLs) and systems biology attempts to assign these regulatory variants to gene regulatory and protein interaction networks (and other omics layers) to infer how regulatory variants perturb biological pathways, thereby linking gene expression changes to downstream cellular functions across key tissues.

To further resolve this complexity, artificial intelligence (AI) algorithms and machine learning (ML) models are increasingly used to sift through and analyze high‐dimensional multi‐omics and multimodal datasets [53]. These tools uncover gene–gene and gene–environment interactions, stratify patients, and predict functional impacts and disease progression [54]. Algorithms such as XGBoost, Random Forests (RF), and ShAP (Shapley Additive Explanations) can identify the most informative features across data layers, for example, highlighting which SNPs, gene expression levels, or pathways influence T2D risk in predictive models. Supervised ML integration of pancreatic islet multi‐omics data, including DNA methylation, transcriptomics, SNP genotypes, and clinical phenotypes, achieved an approximately 91% prediction accuracy in distinguishing T2D patients from controls, significantly outperforming single‐omics models [52]. This integrative model identified key molecular features associated with T2D, such as differential DNA methylation, altered gene expression, and a risk SNP in ANO1, a calcium‐activated chloride ion channel expressed in β‐cells. Its role in regulating membrane depolarization and insulin‐stimulated glucose release highlights the value of cross‐layer multi‐omics analyses.

Explainable AI (XAI) further enhances interpretability by ranking the predictive value of each biomarker, enabling transparent clinical decision‐making. XAI‐based models can accurately predict early insulin resistance in children by integrating genomic, epigenomic, and clinical data. Such pipelines facilitate personalized risk assessment and early intervention [55].

Systems biology and AI together offer a powerful data‐driven approach to resolving the functional bottleneck, forming a pipeline from genetic variants to therapeutic intervention. AI models integrate and analyze multi‐omics data to identify candidate genes, biomarkers, and predictive models, while systems biology contextualizes these findings within biochemical pathways, prioritizing the most influential targets and pathways for intervention.

This represents a significant leap in functional interpretation, transforming abstract genetic loci into concrete genes and pathways where they are present and active. By linking variants to genes, metabolites, and clinical traits, an interconnected map of T2D pathogenesis can be developed [56]. Through this network, it becomes possible to map functionally impactful gene variants by navigating through the omics layers [57].

The synergy of AI and systems biology has already yielded translational outcomes, including the development of biomarker panels that can be used to stratify patients or predict disease progression. Such biomarkers have the potential to be developed into diagnostic tests [58]. These integrative approaches also extend to the gut microbiome, where ML has identified microbial markers of dysglycemia [59, 60]. Deep learning models trained on genomic and epigenomic data have uncovered regulatory modules associated with IR [61, 62]. For example, one module enriched in adipocyte progenitor cells and immune cells was negatively associated with insulin sensitivity, whereas another, enriched in mature healthy adipocytes, correlated positively, highlighting tissue‐specific susceptibilities and therapeutic opportunities [63].

Ultimately, the convergence of AI and systems biology provides a data‐driven framework for resolving the functional bottleneck in T2D. Through AI‐driven multimodal platforms capable of real‐time learning, dynamic patient stratification, and individualized treatment response prediction, this paradigm supports the classification of T2D into mechanistically defined subtypes and accelerates the development of precision therapies, including gene editing and RNA interference, precision nutrition, and microbiome modulation [57].

The future of precision medicine in T2D lies in such integrative, temporal, and AI‐enhanced machine learning frameworks [53, 64]. Large‐scale longitudinal datasets integrating genetic, clinical, and environmental data will further unravel the complex architecture of T2D [65, 66, 67]. These advances promise to transform metabolic disease management, shifting the paradigm from reactive treatment to proactive, precision, and preventative care [68].

## Biochemical pathways driving T2D pathogenesis

**Fig. 2:**
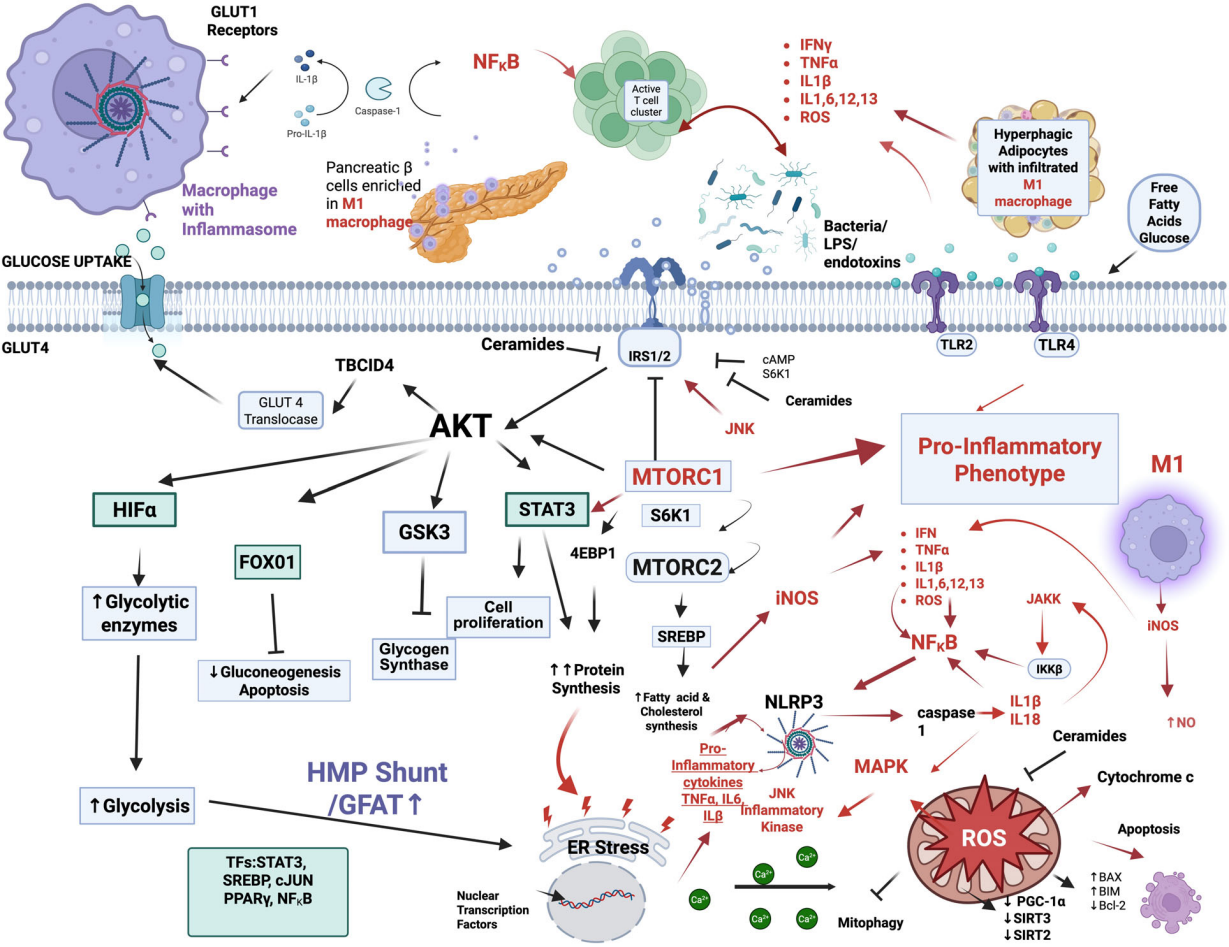
Biochemical pathways underpinning T2D pathophysiology. The AkT insulin signaling pathway is initiated by insulin binding to insulin receptors and activation of insulin receptor substrates (IRSs). Downstream effects include the promotion of glucose uptake (GLUT4 transporters), as well as increased phosphorylation of key intermediates, increased glycolysis, and increased production of glycolytic enzymes and transcription factors. Excess glucose uptake is accompanied by AkT signaling and activation of MTORC 1, driving macrophage differentiation and polarization toward M1 and the pro‐inflammatory phenotype. Increased uptake of glucose by M1 macrophages drives glycolysis and ATP production for cytokine production and release of 4EBP1 (elF4E binding protein 1). 4EBP1binds to elF4E and prevents it from initiating translation. mTORC1 directly phosphorylates 4EBP1, releasing eLF4E (eukaryotic initiation factor 4E), allowing it to participate in the formation of the elF4F complex, which recruits ribosomes to mRNA, initiating translation. This increases the synthesis of proteins essential for cell growth and proliferation. SREBP (sterol regulatory element‐binding proteins) are transcription factors that regulate genes involved in cholesterol and lipid biosynthesis. mTORC1, through activation of SREBP, promotes its translocation to the nucleus, where it upregulates lipid biosynthesis gene expression. The transcription factor HIFα (hypoxia‐inducible factor) regulates nuclear genes involved in glycolysis and angiogenesis. mTORC1 stabilizes and activates HIFα under nutrient‐rich conditions, promoting a shift in metabolism from oxidative phosphorylation to glycolysis in low‐oxygen environments. The transcription factor STAT3 (signal transducer and activator of transcription 3) is activated by cytokines and growth factors, promoting cell survival, proliferation, inflammation, and gene expression related to glucose metabolism and cell survival. The transcription factor FOXO1 promotes gluconeogenesis in the liver. Insulin Akt signaling phosphorylates and inhibits FOXO1, supporting insulin sensitivity and promoting glucose uptake instead of production. Under nutrient excess, oxidative stress, and inflammation conditions, increased flux through HMP increases demand for protein synthesis and glycosylation, interfering with normal processing and folding of glycoproteins in the ER. Misfolded and unfolded proteins accumulate in the ER lumen, triggering the unfolded protein response (UPR), restoring ER homeostasis by reducing protein load and degrading misfolded proteins. ER stress generates ROS and dysregulated calcium release, which leaks into mitochondria, thereby disrupting mitochondrial function, causing oxidative stress, impairing ATP production, and promoting proton and electron leakage, ROS, oxidative stress, and eventual death by apoptosis. Cytochrome c release from mitochondria drives caspase‐mediated apoptosis. ER stress alters the activity of Bcl‐2 proteins in mitochondria and the ER, further promoting apoptosis and cell death. Pancreatic β‐cells become infiltrated with M1 macrophages, driving inflammation. β‐cell stress arises due to the increased demand for extra insulin to meet the hyperglycaemic load. LPS and endotoxins further drive the pro‐inflammatory phenotype by interacting with TLR receptors on the cell membrane. Created in BioRender.

T2D arises from a complex interplay of genetic, metabolic, and inflammatory factors, converging on insulin resistance (IR) and pancreatic β‐cell dysfunction. At the core of these processes lie several interwoven biochemical pathways that regulate glucose and lipid metabolism, cellular energy homeostasis, and immune responses (Fig. 2). The glycolytic pathway, considered the central spine of glucose metabolism, is tightly regulated by insulin signaling through the phosphoinositide 3‐kinase (PI3K)/Akt pathway, facilitating glucose uptake via GLUT4 transporters. However, chronic metabolic stress leads to dysregulation of key pathways, including mammalian target of rapamycin (mTOR), AMP‐activated protein kinase (AMPK), c‐Jun N‐terminal kinase (JNK), and the hexosamine biosynthetic pathway (HBP). These pathways meticulously moderate insulin action, β‐cell survival, and immune cell metabolism, establishing a feedback loop between inflammation, oxidative stress, and IR.

Metabolic signatures of T2D reflect a state of cellular stress in which excess fatty acids, amino acids, and glucose drive hyperglycemia, IR, and β‐cell dysfunction (Fig. 1). In response to metabolic overload, pancreatic β‐cells exhibit excessive insulin secretion, dysregulated potassium signaling, and increased calcium uptake, exacerbating metabolic imbalances. Mitochondrial oxidative stress plays a critical part as increased ATP production to meet energy demands results in electron leakage from the electron transport chain (ETC), generating reactive oxygen species (ROS). These ROS then induce endoplasmic reticulum (ER) protein misfolding, calcium leakage, and oxidative damage, further impairing β‐cell function and increasing apoptosis. In response to nutrient overload, the mTORC1 pathway becomes hyperactivated, promoting glycolysis and shunting glucose into the HMP pathway while simultaneously suppressing AMPK signaling, a key regulator of cellular energy balance. Concomitantly, activation of the MAPK and NF‐κB pathways amplifies pro‐inflammatory cytokine production (e.g., TNF‐α, IL‐6, IL‐1β) and promotes macrophage infiltration in pancreatic islets and adipose tissue, worsening systemic inflammation. Beyond these central pathways, emerging evidence highlights the influence of sirtuins and the gut microbiome in influencing T2D pathophysiology. Such metabolic signatures of T2D highlight a dynamic interplay between nutrient overload, mitochondrial stress, immune activation, and dysregulated insulin signaling. An in‐depth understanding of these pathways provides fundamental insights into the development of targeted therapeutic strategies aimed at restoring insulin sensitivity, preserving β‐cell function, and mitigating inflammation‐driven metabolic stress.

## Insulin and the mTOR signaling pathway

Insulin influences almost every organ, including adipose tissue, liver, muscle, brain, kidneys, and vasculature. Its pulsatile release is critical for effective glucose regulation—periodic insulin pulses help the liver and peripheral tissues respond efficiently and maintain insulin receptor sensitivity. A hallmark of early T2D pathogenesis is the loss of pulsatile insulin secretion. Once secreted, insulin binds to its receptor tyrosine kinases (RTKs) on the surface of target tissues, such as hepatocytes, myocytes, and adipocytes [3]. This initiates phosphorylation events that trigger the PI3K/AkT (protein kinase B) signaling pathway, which facilitates glucose transport into cells through GLUT4 transporters translocating to the plasma membrane [69, 70].

As an integral component of the PI3K/AkT signaling cascade, mTOR acts as a nutrient sensing hub that regulates glucose metabolism, protein synthesis, lipid homeostasis, and cell survival via downstream effectors like S6 Kinase 1(S6K1) and eukaryotic translation initiation factor 4E‐binding protein (4E‐BP). mTORC1 signaling supports β‐cell mass and function by promoting cell growth, proliferation, and survival in response to nutrients [71]. However, sustained mTORC1 activation (such as in overnutrition) increases S6K1 activity, which feeds back to impair insulin signaling, thereby contributing to the development of IR [72, 73, 74]. Maintaining optimal β‐cell mass is essential for correct insulin production and metabolic regulation [75] (Fig. 2). In parallel, mTOR complex 2 (mTORC2) regulates glucose metabolism through both Akt‐dependent and independent mechanisms. mTORC2 activity is enhanced under reduced amino acid availability or glucose deprivation as part of the cellular adaptation to nutrient scarcity. Conversely, mTORC2 dysregulation contributes to mitochondrial dysfunction, oxidative stress, and lipid accumulation, which exacerbate β‐cell function and IR [76].

Beyond its metabolic role, mTOR signaling influences immune cell differentiation and function, contributing to the inflammatory state that drives T2D. T cells, the central regulators of immunity, undergo metabolic reprogramming upon activation, shifting from oxidative phosphorylation to glycolysis to meet the heightened energy and biosynthetic demands of an immune response. This metabolic shift, mediated by mTOR signaling and Myc, upregulates nutrient transporters, such as GLUT1 and GLUT4, to facilitate glucose uptake for rapid energy generation and macromolecule synthesis. Similarly, antigen‐presenting dendritic cells undergo a metabolic shift toward mTOR‐driven glycolysis and activate the transcription factor STAT3 (signal transducer and activator of transcription 3), metabolic changes that enhance their migration to lymph nodes and promote T cell activation. These shifts influence dendritic cell subsets, shaping T cell polarization (e.g., Th1, Th2, Th17, or Treg), which is crucial for tailored immune responses to pathogens and disease states.

CD8^+^ and CD4^+^ T cells work in unison to damage and kill β‐cells, with active CD4^+^ T cells differentiating into helper (Th1, Th2, Th17) and regulatory (Tregs) subsets. Pro‐inflammatory cytokines (e.g., IFNγ, TNF‐α, IL‐1β) from Th1 and Th17 drive β‐cell destruction in T1D, while anti‐inflammatory cytokines (e.g., IL‐10, TGF‐β) from Th2 and Tregs protect β‐ cells by suppressing inflammation and promoting tolerance to self‐antigens. Tregs maintain immune tolerance to pancreatic β‐cell antigens and suppress autoreactive T cells. Balance among T cell subsets is crucial for immune homeostasis. Disruption of this balance leads to islet inflammation and may contribute to β‐cell stress in T2D as well [77, 78].

Macrophages adopt polarized phenotypes: M1 (pro‐inflammatory) and M2 (anti‐inflammatory). Pro‐inflammatory cytokines such as IL‐1β, TNF‐α, and pathogen‐associated molecular patterns (PAMPs) stimulate cell surface receptors such as Toll‐like receptors (TLRs) and respond to endoplasmic reticulum (ER) stress. IL‐1β binds to interleukin 1 receptor type I (IL‐1R1), triggering a downstream intracellular signaling cascade activating JAK protein kinases and the IKK complex. Phosphorylation of IRS substrates by IKK and JNK leads to inhibition of insulin signaling [79]. IKK phosphorylates IκB, leading to its ubiquitination and degradation by the proteasome, releasing NF‐κB and allowing it to translocate into the nucleus and promote early expression of anti‐apoptotic and pro‐inflammatory genes. Elevated IL‐1β levels have been shown to increase the risk of T2D. Mice with constitutively active NF‐κB in β‐cells develop insulitis and immune‐mediated diabetes. In a pro‐inflammatory environment, M1 macrophages overexpress GLUT 1 transporters to uptake glucose and fuel anaerobic glycolysis, supporting rapid production of pro‐inflammatory cytokines (IL‐1β, TNF‐α, IL‐1, IL‐ 6, IL‐12, IL‐23), nitric oxide (NO) [80], ROS, and inducible nitric oxide synthase (iNOS). Together, T cells and M1 macrophages drive chronic inflammation in metabolic tissues. They accumulate in the adipose tissue of obese mice and in the liver and pancreas of mice with IR [81] (Fig. 2).

Anti‐inflammatory M2 macrophages, supported by mTORC2/PI3K/Akt signaling, promote tissue repair and immune tolerance. M2 macrophages rely on oxidative metabolism for energy production. Induced by anti‐inflammatory signals such as IL‐4, IL‐6, IL‐10, IL‐13, or glucocorticoids, M2 macrophages secrete anti‐inflammatory cytokines, including IL‐10 and TGF‐β. Both Tregs and M2 macrophages are enriched in the adipose tissue of lean mice and in the healthy tissue of insulin‐responsive animals [82]. The integration of mTOR signaling within the insulin signaling pathway emphasizes its essential role in coordinating glucose metabolism, β‐cell function, and immune regulation. While transient activation of mTORC1/mTORC2 is necessary for normal insulin action, dysregulation of these complexes promotes IR, metabolic dysfunction, and inflammation, accelerating T2D progression.

## AMP kinase signaling

AMP‐activated protein kinase (AMPK) functions as a master energy sensor within the cell. The cellular AMP:ATP ratio is critical in regulating AMPK activity and protecting cells against ATP depletion [83]. In response to energy starvation or physiological cellular stress (e.g., heat, oxidative stress, glucose scarcity and/or the consumption of ATP through exercise), a high AMP/ATP ratio allosterically activates AMPK, which shifts metabolism toward catabolism and decreased anabolism through the phosphorylation of key enzymes in metabolic pathways [84]. Upstream kinases in pathways involved in ATP production include liver kinase B1 (LKB1), calcium/calmodulin‐dependent protein kinase (CAMKKβ) and the transforming growth factor β‐activated kinase 1 (TAK1). These kinases regulate key processes such as mTORC1 signaling, glycolysis, lipid homeostasis, mitochondrial function, and mitophagy [85]. Direct phosphorylation of AMPK by CAMKKβ and activation (independent of LKB1 and AMP levels) in response to intracellular calcium flux links calcium signaling to the regulation of energy metabolism. Activated AMPK improves glucose uptake into muscle through either cross talk with Akt or by increasing GLUT4 expression and translocation to the plasma membrane. AMPK stimulates clearance of intramyocellular fat through β‐oxidation, thus improving insulin sensitivity. In the liver, AMPK activation promotes fatty acid oxidation and reduces synthesis of cholesterol through inhibition of HMG‐CoA reductase. As a central regulator of metabolism, AMPK acts at cellular and physiological levels to circumvent metabolic stress by restoring energy balance [85, 86, 87].

## The JNK signaling pathway: The junction between insulin resistance and inflammation

Mitogen‐activated protein kinases (MAPKs) and Janus kinases (JAKs) are critical mediators of oxidative stress responses and inflammation. MAPKs, including ERK, JNK, and p38, are activated by ROS, leading to phosphorylation of transcription factors such as NF‐κB and AP‐1. This drives production of pro‐inflammatory cytokines like TNF‐α, IL‐6, and IL‐1β, contributing to chronic inflammation [88]. JNK is regarded as a stress response “inflammatory” kinase that also regulates apoptosis and metabolic reprogramming. Upon activation, JNK phosphorylates several nuclear transcription factors (e.g., cJUN, ATF2 and SP1), inducing the transcription of target genes such as *NCOR1* that propagate IR and inflammation. JNK also phosphorylates certain non‐nuclear proteins including mitochondrial BCL‐2 family members (BCL‐XL, BCL‐2), thereby influencing cell growth, differentiation, proliferation, cell survival, and metabolic stress pathways [89, 90, 91]. The JAK/STAT pathway is activated by oxidative stress, resulting in STAT phosphorylation, dimerization, and nuclear translocation, which initiate inflammatory gene expression [92]. Overnutrition and obesity heighten systemic inflammation through adipokines and free fatty acids (FFAs), promoting macrophage infiltration and secretion of cytokines like TNF‐α and IL‐1β. TNF‐α activates JNK, which phosphorylates IRS1/2, disrupting PI3K/Akt signaling and impairing insulin action. JNK also promotes pro‐inflammatory cytokine gene expression in macrophages, such as IL‐6, through nuclear transcription factors (e.g., c‐JUN, ATF2, ELK1) [93]. FFAs further activate NF‐κB, creating a feedback loop that amplifies cytokine production (including IL‐6) and exacerbates inflammation [94]. Sustained JNK activation, due to elevated FFAs, negatively impacts insulin secretion and causes β‐cell dysfunction and death [95] (Fig. 2).

## The hexosamine biosynthetic pathway and mTOR/AMPK cross talk

Under physiological conditions, 1–3% of intracellular glucose is shunted from the glycolytic pathway to the hexosamine biosynthesis pathway (HBP) [96]. The resulting hexosamines (e.g., UDP‐N‐acetyl glucosamine ‐UDP‐GlcNAc) are used for glycosylation of proteins, lipids, and nucleic acids, thereby modulating their activity, stability, and function. Hexosamines can be generated via *de novo* or salvage pathways and require nutrients such as glutamine, glucose, acetyl‐CoA, and UTP. *De novo* synthesis of UDP‐N‐acetyl glucosamine (UDP‐GlcNAc) through the HBP is catalyzed by the rate‐limiting enzyme glutamine: fructose‐6‐phosphate amidotransferase (GFAT). GFAT1 interacts with mTORC2 and PTEN in the PI3K/Akt signaling pathway. PTEN's lipid phosphatase activity opposes PI3K by dephosphorylating PIP3, thus dampening insulin signaling. PTEN's interaction with GFAT suggests that cellular nutrient status might modulate insulin sensitivity via PTEN and downstream PI3K/Akt/mTOR signaling [97, 98]. Cross talk between GFAT, PTEN, and mTORC2 could help maintain cellular homeostasis by appropriately responding to nutritional cues [99] (Fig. 2).

P13K/mTOR signaling can upregulate HBP flux during nutrient excess, whereas glucose deprivation or inhibition of glycolysis enhances mRNA and protein expression of *GFAT1* as a compensatory response [96, 100]. During prolonged starvation or heightened cellular demand, AMPK phosphorylates GFAT1, diminishing its enzymatic activity, leading to a decrease in N‐linked protein glycosylation. Elevated HBP activity correlates with key markers of T2D severity such as HbA1c, IR, postprandial plasma glucose levels, and oxidative stress indicators (protein carbonyls and lipid peroxidation). Under conditions of overnutrition, hyperglycemia, and hyperinsulinemia, this cross talk is skewed toward high HBP flux and mTORC1 activation, with inadequate AMPK counterbalance resulting in a biochemical milieu that favors IR, postprandial hyperglycemia, and oxidative stress. The pathological consequences of HBP hyperactivation extend to vascular complications in T2D, which include diabetic nephropathy, delayed wound healing, cardiac hypertrophy, and heart failure. As GFAT can influence both energy sensing (AMPK) and growth‐sensing (mTOR) axes that are dysregulated in T2D, it serves as a promising intervention point for therapy [101]. Pharmacological inhibitors such as 6‐diazo‐5‐oxo‐LL‐norleaucine (DON) and O‐diazoacetyl‐L‐serine (azaserine) that mimic glutamine have been shown to suppress GFAT activity and demonstrate efficacy in reversing diabetic phenotypes in preclinical models. However, their use is limited due to toxic effects on the gastrointestinal tract. AMPK activation through the use of drugs like metformin in muscle and liver not only represses gluconeogenesis but can also indirectly reduce HBP activity. While mTORC1 inhibitors (e.g., rapamycin) have been shown to reduce high glucose‐mediated IR by blocking the mTOR/S6K‐mediated feedback on IRS1, chronic mTOR inhibition can adversely affect β‐ cell growth and lead to immune suppression. In pancreatic β‐cells, moderate HBP flux and O‐GlcNAc glycosylation have been shown to support cell survival and insulin secretory function during the early stages of T2D. Conversely, loss of HBP activity results in mTORC1 activation and autophagy. Given that HBP intersects with fundamental cellular processes, such as ER‐associated glycosylation, and while it is a pivotal pathway in nutrient excess conditions, fine‐tuning rather than complete inhibition has the potential to control flux through the pathway. Emerging evidence supports combinatorial therapies that incorporate GFAT inhibitors with established anti‐diabetic drugs. For instance, pairing a low‐dose GFAT inhibitor with metformin or a glucagon‐like peptide‐1 receptor agonist (GLP‐1 RA) could synergistically restore metabolic homeostasis by correcting nutrient signaling and enhancing insulin sensitivity. Development of inhibitors that mimic UDP‐GlcNAc feedback inhibition may offer an alternative approach to regulate GFAT‐1 activity, while PKA‐mediated phosphorylation of GFAT‐1 is another option for therapeutic intervention. Importantly, understanding the isoform‐specific functions and tissue‐specific expression of GFAT will be central to the development of precision‐targeted therapies [102]. Such interventions aim to preserve beneficial glycosylation processes required for cellular function while mitigating pathological hyperactivation of HBP associated with metabolic dysfunction.

## Sirtuins: Multifunctional regulators of inflammation, metabolism, and genomic stability

The sirtuin family (SIRT1‐7) consists of NAD^+^‐dependent deacetylases that regulate inflammation, metabolism, and genomic stability. Many studies report on the mechanisms by which the SIRT family participates in inflammation, especially pathways involving NFκB, TNF‐α, and the NLRP3 inflammasome [103, 104, 105]. Most SIRTs (SIRTs 1, 2, 6 and 7) exert anti‐inflammatory effects through NFκB and its pathway by preventing its nuclear translocation and expression, and/or regulating its interactions.

Beyond immunity, sirtuins significantly influence metabolic pathways. SIRT1, SIRT3, and SIRT6 regulate glucose and lipid metabolism. SIRT1 improves insulin sensitivity and glycemic control by deacetylating LKB1, promoting its translocation from the nucleus to the cytoplasm, thereby upregulating AMPK. AMPK and SIRT1 are interconnected components of cellular energy sensing and metabolic regulation [106]. AMPK and SIRT1 can reciprocally activate each other: AMPK activation increases cellular NAD^+^ levels, which in turn activate SIRT1. This interplay modulates metabolic pathways, including mitochondrial biogenesis and energy expenditure. SIRT1 also inhibits hepatic gluconeogenesis by suppressing key gluconeogenic enzymes (PEPCK, G6Pase, and coactivator PGC‐1α), while promoting glycogen storage.

SIRT6 maintains glucose homeostasis through deacetylation of FOXO1 and upregulation of GLUT2 in pancreatic β‐cells, as well as regulation of insulin secretion and systemic glucose tolerance. Improvements in SIRT6‐mediated insulin signaling have been reported in the liver of obese rats after exercise [107]. SIRT2 prevents hepatic steatosis by deacetylating hepatocyte nuclear factor 4α (HNF‐4α). SIRT5, primarily studied in adipose and liver tissue, activates AMPK in obese mice. SIRT5 activation inhibits pre‐adipocyte differentiation and lipid deposition and suppresses MAPK signaling, thereby reducing inflammation in adipose tissue [108].

In addition to metabolic regulation, sirtuins protect against oxidative stress and support cellular housekeeping. SIRTs 1, 3, and 6 enhance antioxidant defenses and maintain mitochondrial function in β‐cells [105, 109]. SIRT1 improves cellular antioxidant capacity by deacetylation and activation of key transcriptional regulators like FOXO1 and Nrf2, which in turn increase the expression of antioxidant enzymes (SOD2 and catalase) and anti‐apoptotic proteins (p53 and KU70). SIRT3 drives mitochondrial biogenesis by deacetylating key enzymes, enhancing their activity. It supports efficient respiration, activating TCA cycle dehydrogenases and components of the ETC, enhancing ATP production and minimizing ROS leakage. SIRT3‐null mice show reduced oxygen consumption and increased ROS production.

SIRT3 also regulates β‐oxidation and amino acid metabolism [110]. SIRT2 regulates mitochondrial protein deacetylation, mitophagy, and mitochondrial function. SIRT2 deficiency leads to hyperacetylation of mitochondrial proteins, morphological mitochondrial changes, increased ROS, and diminished ATP production, with impaired mitophagy leading to the accumulation of dysfunctional mitochondria. Sirtuins interface with master regulators like PGC‐1α (a coactivator of PPARδ/γ) to mitigate oxidative stress. SIRT1 and SIRT3 activity enhance PGC‐1α induction of antioxidant enzymes and mitochondrial function and biogenesis, thereby enhancing scavenging of excess ROS and maintaining energy homeostasis [111].

Under cellular stress, SIRT1 promotes autophagy by deacetylating core autophagy proteins (Atg5, Atg7, and Atg8) and by targeting cytoplasmic p53 for degradation through activation of ubiquitin E3 ligase Mdm2. SIRT1 further supports autophagy and energy balance by deacetylating LKB1 (upstream of AMPK) and facilitating its translocation from the nucleus to the cytoplasm, thereby activating AMPK [110].

Cancer and T2D share profound convergence across multiple metabolic and signaling pathways [112]. These include metabolic reprogramming, chronic inflammation, oxidative stress, and dysregulated growth and survival signaling, reflecting a shared pathophysiological architecture. Metabolic reprogramming, one of the hallmarks of cancer, is increasingly recognized in the early pathogenesis of T2D. In insulin‐resistant tissues such as the liver and adipose, mitochondrial dysfunction and a compensatory shift toward glycolysis mirror the Warburg effect observed in tumors. In both conditions, metabolic stress activates inflammatory cascades such as NF‐κB and NLRP3 inflammasome activation, while lipotoxic intermediates like ceramides further compromise mitochondrial function and promote ER stress. Cancer cells, however, develop mechanisms to circumvent ceramide‐induced apoptosis by increasing glucose uptake, thereby favoring aerobic glycolysis. This supports rapid ATP production for proliferation, and it also creates an acidic microenvironment that enhances immune evasion and promotes angiogenesis. Similarly, in insulin‐resistant tissues such as the liver and adipose, early T2D is characterized by mitochondrial dysfunction, a shift toward glycolysis, and excess production of ROS, which collectively impair insulin signaling and β‐cell viability.

In cancer, ceramide metabolism is often dysregulated, with many tumors developing mechanisms to resist ceramide‐induced apoptosis through upregulation of glucosylceramide synthase or alterations in sphingolipid pathways. Apoptotic resistance is another shared feature. While T2D β‐cells succumb to ER and oxidative stress, tumor cells evade apoptosis through mutations in p53 and *PTEN*. Epigenetic reprogramming is also observed in both diseases, driving IR in T2D and oncogene activation in cancer.

Within this overlapping terrain, SIRT1 emerges as a dual regulator of metabolic and survival pathways. SIRT1 represses NF‐κB signaling, inhibits hepatic lipogenesis through SREBP‐1c, and promotes insulin sensitivity via activation of PGC‐1α and FOXO1. However, its interactions with transcription factors such as p53, E2F1, and the tumor suppressor protein hypermethylated in cancer 1 protein (HIC1) reveal a complex oncogenic potential. While SIRT1 deacetylates and stabilizes metabolic regulators, it also deacetylates p53, thereby reducing its tumor‐suppressive function. Loss of HIC1 leads to upregulation of SIRT1 expression, promoting oncogenesis. Moreover, SIRT1‐mediated deacetylation of E2F1 suppresses its transcriptional activation of pro‐apoptotic genes, shifting the cellular response toward survival.

Similarly, SIRT3, a mitochondrial sirtuin, suppresses ROS and preserves mitochondrial function, conferring metabolic advantages in T2D. Yet, in cancer, this redox adaptation can inadvertently support tumor survival. Immunosuppression, as a result of SIRT1 and SIRT3 activation, can reduce immune surveillance, thereby promoting cancer progression. Therefore, systemic activation of sirtuins presents a therapeutic paradox: while beneficial for reversing T2D pathology, it could also reduce immune surveillance and promote tumor growth.

Targeted delivery and patient stratification are therefore essential. Tissue‐specific delivery vectors (e.g., liposomes, viral carriers) should direct SIRT modulation toward insulin‐responsive tissues such as liver, muscle, and adipose tissue to minimize oncogenic risk. Development of isoform‐selective modulators further enables fine‐tuning of SIRT1 and SIRT3 activity [113] A multi‐omics framework can aid in patient stratification. Integration of genomic data (e.g., SNPs in *SIRT1*, *SIRT3*, *PGC‐1α*), transcriptomic signatures (e.g., inflammatory and proliferative gene expression), and metabolomic profiles (e.g., NAD^+^/NADH ratio, redox metabolites) can identify individuals at high cancer risk.

In such patients, sirtuin‐based interventions may be best combined with mTOR inhibitors, p53 stabilizers, or immune checkpoint inhibitors to offset oncogenic potential [114]. Ultimately, sirtuins are uniquely positioned as therapeutic integrators of metabolic, inflammatory, and mitochondrial dysfunction in T2D. However, their successful clinical application requires a pathway‐specific, omics‐informed framework that balances therapeutic efficacy against oncogenic liability [115].

## The gut microbiome

The human gut contains the majority of the body's microbiome, including at least 1000 distinct species of bacteria, and encodes approximately 150 times the number of genes as the human genome. Coevolution between the human host and gut microbiota has established a mutually beneficial symbiotic relationship, whereby the host provides nutrients and a suitable environment, while the microbiota contribute to host homeostasis via metabolic regulation, immune modulation, and protection against pathogens. Imbalances in the gut microbiota ecosystem termed *dysbiosis* are associated with many metabolic disorders, including obesity and T2D [116].

Diet plays a central role in shaping the gut microbiome, as specific nutrients can selectively promote or inhibit the growth of particular microbial taxa. While environmental factors such as diet, medications (e.g., metformin, proton pump inhibitors), and lifestyle are the dominant drivers of gut microbial diversity, host genetics also contribute significantly to microbiome composition [117]. GWAS and twin studies (TwinsUK cohort) have demonstrated genetic variation influences the relative abundance of specific microbial taxa and the overall structure of the gut microbiome [118, 119]. Both human and mouse studies have demonstrated that a subset of gut bacteria is heritable and influenced by host genetics [117, 118, 120]. For instance, polymorphisms in the *FUT2* (Fucosyltransferase 2) gene affect the fucosylation of intestinal mucins, thereby impacting microbial colonization and influencing downstream microbial energy metabolism and mucosal inflammation [121]. Similarly, polymorphisms in the *LCT* lactase gene, notably rs4988235, determine lactase persistence (LP) or non‐persistence (LNP), significantly influencing the relationship between dairy intake and T2D risk. In individuals with the LNP genotype, higher milk consumption is associated with a significantly reduced risk of T2D. This protective effect is driven by shifts in gut microbiota characterized by an increase in the abundance of lactose‐fermenting *Bifidobacterium* and a reduction in *Prevotella* species, taxa that are associated with improved metabolic health [122]. These microbial changes align with favorable alterations in circulating metabolites, including elevated levels of the anti‐inflammatory compound indolepropionate and reduced levels of branched chain amino acids (BCAAs), both of which are known to enhance insulin sensitivity and reduce systemic inflammation. Together, these findings support a genotype‐dependent, microbiota‐mediated mechanism through which dairy intake modulates T2D risk.

Further evidence of host–microbiome cross talk comes from studies on tryptophan metabolism [123]. In mice, genetic inhibition of indoleamine 2,3‐dioxygenase (IDO) shifts tryptophan catabolism from the host kynurenine pathway toward microbial production of indole derivatives, thereby improving insulin sensitivity. In humans, higher circulating plasma levels of tryptophan and kynurenine pathway metabolites (kynurenate and xanthurenate) are associated with increased T2D risk, whereas higher levels of microbial‐derived metabolite indolepropionate are associated with reduced risk [124].

Although much of the interindividual variability in gut microbiota is environmentally driven, host genetic loci involved in immune regulation, insulin signaling, and glucose metabolism have been associated with shifts in microbial communities that promote metabolic dysfunction [125]. For example, individuals carrying specific variants of the PPARγ coactivator 1 alpha‐2 (*PPARγC1*‐α) gene exhibit reduced gene expression, a shift in gut microbial composition, with an enrichment of *Prevotella copri*, *Megasphaera*, and *Bacteroides vulgatus* taxa implicated in IR in humans, and capable of inducing IR in murine models. Variants in *TCF7L2* have been associated with disrupted microbial fermentation profiles and a loss of butyrate‐producing taxa such as *Roseburia* [126].

Variations in gut microbiome composition also account for up to 6% of the variance in circulating lipid levels among healthy individuals. An unfavorable lipid profile, low HDL cholesterol, elevated triglycerides, and increased BMI have been associated with higher abundance of *Eggerthella* and *Blautia* and decreased levels of *Akkermansia*, *Rikenellaceae*, and *Christensenellaceae* [119].

Microbiome dysbiosis has been implicated in key pathophysiological features of T2D, including metabolic endotoxemia, chronic inflammation, and impaired glycemic control. T2D‐associated microbiomes exhibit decreased species diversity, an overrepresentation of facultative anaerobes, and depletion of beneficial butyrate‐producing obligate anaerobes. Functionally, the T2D gut microbiome is enriched in pathways related to carbohydrate transport, fatty acid and BCAA metabolism, and xenobiotic degradation, features reflective of a dysregulated microbial ecosystem contributing to disease progression [119].

Gut microbiota ferment non‐digestible dietary components in the colon into metabolites— short‐chain fatty acids (SCFA: acetate, propionate, butyrate) that contribute roughly 5–10% of daily human energy needs. SCFAs help maintain gut epithelial integrity and regulate β‐cell proliferation and insulin secretion [127]. Butyrate, in particular, affects intestinal macrophage activity, downregulates the production of pro‐inflammatory mediators (e.g., LPS induced IL‐6 and IL‐12) and promotes the differentiation of anti‐inflammatory Tregs [128]. Dysbiosis reduces SCFA production, increasing gut permeability and inflammatory mediators (e.g., histone deacetylase 3, ROS and IL‐1β), while reducing anti‐inflammatory mediators (e.g., IL‐10 and IL‐17), thereby promoting IR and systemic inflammation. Increased gut permeability facilitates the translocation of microbial products like lipopolysaccharide (LPS) and other metabolites into circulation, leading to metabolic endotoxemia. High‐fat diets worsen gut permeability and activate TLR2/TLR4 signaling, pattern recognition receptors predominantly expressed on macrophages, thus further amplifying inflammation [129, 130] (Fig. 1).

Beyond SCFAs, gut microbes influence host metabolism through a variety of other bioactive compounds. Certain intestinal bacteria (such as *Akkermansia muciniphila*) generate metabolites (like 2‐oleoyl glycerol, 2 palmitoyl glycerol, and 2 acyl glycerol) derived from dietary fats. In adipose tissue, such metabolites can promote adipocyte differentiation and fatty acid oxidation by downregulating PPARγ activity [131].

Branched chain amino acids (leucine, isoleucine, valine) are often elevated in serum metabolomes of patients suffering from obesity, IR, or T2D, serving as biomarkers for disease risk. Gut microbiota, such as *Prevotella copri* and *Bacteroides vulgatus*, can synthesize BCAAs, contributing to these elevated levels exacerbating IR [132]. Excess leucine activates mTORC1, inducing ER stress and β‐cell dysfunction [133]. Conversely, microbial tryptophan metabolites, such as indoleacetate, indolelactate, and indolepropionate, strengthen intestinal epithelial barrier function, stimulate insulin secretion, and enhance glucose homeostasis. Other microbial metabolites, such as 4‐cresol produced by *Clostridia* species from tyrosine, have been shown to reduce adiposity, enhance β‐cell proliferation, and improve glucose tolerance by mechanisms similar to GLP‐1 RAs [134, 135].

Certain gut microbes actively prevent inflammation by inducing the production of anti‐inflammatory cytokines such as IL‐10 and IL‐22. *Bacteroides fragilis* and *Roseburia intestinalis* improve glucose metabolism and insulin sensitivity in muscle tissue. Other commensals enhance Treg‐associated gene expression, promote the differentiation of T cells into Tregs, and induce the production of anti‐inflammatory cytokines such as TGF‐β and IL‐10. *Bacteroides thetaiotaomicron*, a key gut symbiont, regulates the activity of Th1/Th2/ Th17, promoting immune homeostasis. Various *Lactobacillus* species (e.g., *L. plantarum*, *L. paracasei*, *L. casei*) suppress the production of pro‐inflammatory cytokines (e.g., IL‐6, IL‐1β, IL‐8) and inhibit TNF‐α, IFN‐γ, and NF‐κB activation, collectively reducing inflammation [136].

Interestingly, many diabetes medications, including metformin, have been reported to disturb the gut microbiota population, an effect that may contribute to their therapeutic efficacy. Metformin has been shown to enrich populations of SCFA‐producing bacteria and *Akkermansia muciniphila*, supporting mucosal barrier integrity and improving insulin sensitivity [137, 138].

## Adipose tissue as an endocrine organ and energy store

Long viewed solely as a lipid storage depot, adipose tissue is now recognized as a central endocrine player in T2D pathophysiology through its secretion of various adipokines, cytokines, chemokines, and growth factors. In states of overnutrition and obesity, hypertrophied adipocytes signal metabolic stress by releasing pro‐inflammatory cytokines, contributing to IR, the hallmark of T2D [139]. Among the key adipokines, leptin and adiponectin function as metabolic homeostatic and appetite‐regulating signals in the body. Leptin acts as a satiety hormone, signaling the hypothalamus to suppress appetite and increase energy expenditure. However, in obesity (a common precursor to T2D), chronically elevated leptin levels lead to leptin resistance, impairing its regulatory effects and promoting further weight gain and IR. Conversely, adiponectin enhances insulin sensitivity, stimulates glucose utilization, and promotes lipid oxidation. Adiponectin levels inversely correlate with fat mass and exist in multiple isoforms (low, medium, and high molecular weight) [140]. Adiponectin exerts its effects through signaling cascades involving AMPK activation and suppression of stress pathways (mTOR, NF‐κB, JNK) with downstream effects on PI3K/Akt and transcription factors like PPARα and STAT3. These actions collectively modulate lipid metabolism, endothelial function, and inflammation—all processes disrupted in T2D [80, 140]. In lean individuals, adipose tissue secretes predominantly anti‐inflammatory adipokines (e.g., adiponectin and secreted frizzled‐related protein‐5) that help maintain insulin sensitivity. In obesity, adipose tissue generates large amounts of pro‐inflammatory mediators, including leptin, resistin, retinol‐binding protein 4 (RBP4), lipocalin 2, and cytokines such as IL‐6, IL‐1β, and TNF‐α. This creates chronic low‐grade inflammation that attracts immune cells (particularly T cells) into adipose depots [141, 142, 143]. The recruited T cells subsequently attract monocytes, which differentiate into pro‐inflammatory M1 macrophages, amplifying inflammation via the NF‐κB pathway, increasing oxidative metabolism, iNOS expression, and ROS production, and worsening IR (Fig. 1). Elevated circulating FFAs, common in obesity, further drive IR and glucose intolerance by interfering with insulin receptor signaling and increasing lipotoxicity in peripheral tissues (muscle, liver) [144, 145]. Chronic inflammation and adipocyte dysfunction, therefore, form a vicious circle linking excess adiposity to T2D pathogenesis (and related conditions like atherosclerosis) [146].

In obesity, pro‐inflammatory M1 macrophages can constitute up to 40% of adipose tissue, exacerbating local inflammation. Conversely, lean adipose tissue is enriched in Tregs, which secrete IL‐10, IL‐4, IL‐13, and TGF‐β, which collectively dampen inflammation and restore metabolic homeostasis.

PPAR (α, γ, and δ) are nuclear receptors central to lipid metabolism, inflammation, and insulin sensitivity. PPAR‐γ, the master regulator of adipogenesis and adipocyte differentiation, is expressed in adipose, skeletal muscle, pancreatic, and immune cells. Hepatic PPAR‐γ influences lipid metabolism through the regulation of genes involved in fatty acid oxidation and triglyceride synthesis. It promotes M2 macrophage polarization, reducing inflammation via NF‐κB repression. Unsaturated FAs and derivatives provided by the diet, *de novo* lipogenesis, and triglyceride lipolysis are natural PPAR ligands that enhance insulin sensitivity and reduce hepatic steatosis and lipogenesis. Polymorphisms in PPAR‐γ have been associated with a reduced risk of developing T2D across populations. Aberrant insulin and leptin signaling in the brain is another key driver of systemic metabolic disease. Impaired hypothalamic signaling, despite high leptin levels, disturbs satiety regulation. For example, IKKβ‐induced activation of NF‐κB in hypothalamic neurons contributes to neuroinflammation, which can interfere with leptin signaling pathways and promote leptin resistance [147].

## Endoplasmic reticulum stress

Biogenesis of secretory proteins, like insulin, begins in the ER through the concerted action of ER‐resident protein folding machinery comprising chaperones, glycosylating enzymes, and oxidoreductases that fold nascent polypeptides and form disulfide bonds. The ER also functions in lipid synthesis and is a major Ca^+2^ storage site, releasing Ca^+2^ in response to signals. Ca^+2^ ions are sequestered in the ER due to the activity of the sarco‐endoplasmic reticulum ATPase (SERCA) pump and are released into the cytosol in response to physiological triggers such as the insulin Akt/P13K signaling pathway, which generates the second messenger IP3.

If demand on the ER protein folding machinery exceeds its capacity, unfolded secretory proteins start to accumulate and the unfolded protein response (UPR) is activated to restore cellular homeostasis. To restore cellular equilibrium, the ER‐associated degradation (ERAD) pathway identifies the defective proteins, extracts them from the ER, and targets them for degradation by the proteasome in the cytosol. Pancreatic β‐cells work close to the limit of protein synthetic capacity, producing more than 1 million insulin molecules/β‐cell/min. Because of their mandate to synthesize and secrete insulin in large and requisite amounts to buffer blood glucose fluxes to maintain normoglycemia, β‐cells have an innate fragility and are susceptible to secretory overload. To compensate for the increasing insulin demand, a significant burden is placed on the ER, where insulin is processed. Elevated levels of saturated FAs cause β‐cells to proliferate and increase insulin synthesis and secretion to maintain normoglycemia. To resolve the stress, maintain β‐cell function, and restore the cell to normal, autophagy is induced to ensure the smooth functioning of UPR machinery and maintenance of ER homeostasis, restoring normal conditions to the cell [148]. Inhibition of autophagy has been shown to promote ER stress‐mediated cell death [149, 150]. Unremitting ER stress activates pro‐apoptotic pathways like CHOP and JNK, culminating in β‐cell apoptosis and contributing to the loss of β‐cell mass in T2D [150, 151].

In addition, the ER is a site of ceramide synthesis. Ceramides are central intermediate molecules in the synthesis of sphingolipids, molecules implicated in metabolic dysfunction. Primarily recognized as lipid bilayer building blocks, ceramides also serve as potent intracellular signaling mediators with profound diabetogenic effects. Ceramides have pleiotropic actions relevant to metabolic disease. Elevated ceramide levels disrupt insulin signaling pathways, induce β‐cell apoptosis, and fuel inflammation, core features of glucolipotoxicity and key pathophysiological hallmarks of T2D [152]. Ceramide accumulation within cellular membranes, including the plasma membrane, ER, mitochondrion, and lysosome, impairs fatty acid metabolism and reduces mitochondrial efficiency through inhibition of Complex I and III of the ETC, which elicits elevated ROS levels and increases membrane permeability, further inducing apoptotic pathways [153]. Ceramides interfere with insulin signaling by activating protein phosphatase 2A (PP2A), leading to dephosphorylation and inactivation of Akt. This cascade inhibits GLUT4 translocation, thereby reducing glucose uptake in insulin‐sensitive tissues such as muscle and adipose tissue. Ceramides also activate NF_κ_B‐mediated inflammatory signaling, which contributes to the pro‐inflammatory state in T2D. One of the key modulators of ceramide biosynthesis is TLR4. TLR4 is activated in response to pathogen‐associated molecular patterns (PAMPs) such as LPS and damage‐associated molecular patterns (DAMPs) including oxidized LDL. While TLR4 signaling in macrophages is important for the development of innate immunity by detecting microbial threats, its chronic activation is implicated in the pathogenesis of several metabolic and inflammatory diseases [154]. TLR4 activation initiates downstream signaling cascades involving NF_κ_B and MAP kinases, resulting in the transcription of pro‐inflammatory cytokines [155, 156]. Moreover, TLR4 signaling is also associated with the induction of enzymes in the *de novo* ceramide biosynthesis pathway. In insulin‐resistant states, this pathway becomes dysregulated, resulting in increased ceramide accumulation that further impairs insulin signaling, perpetuating a vicious cycle of metabolic dysfunction [157]. Therefore, reducing ceramide levels has become a promising therapeutic strategy for metabolic and cardiovascular diseases. SIRT1‐mediated deacetylation of TLR4 promotes its inactivation and degradation [158]. Pharmacological inhibitors of ceramide synthesis such as myriocin and fumonisin B1 target ceramide synthesis directly, reducing intracellular ceramide levels and thereby restoring insulin sensitivity [158]. Preclinical studies have demonstrated that such inhibitors enhance glucose uptake, reduce inflammation, and preserve β‐cell integrity, highlighting their potential as novel therapeutic alternatives to current anti‐diabetic treatments.

The adipokine adiponectin possesses intrinsic ceramidase activity and catalyzes the hydrolysis of ceramides into sphingosine. Sphingosine is in turn phosphorylated by sphingosine kinase to produce sphingosine 1 phosphate (S1P). S1P promotes insulin sensitivity, cell survival, and mitochondrion function. Increases in the S1P to ceramide ratio have been shown to ameliorate endothelial dysfunction through increasing NO levels. Adiponectin receptors (AdipoR1 and AdipoR2) activate primary downstream signaling pathways, including the AMPK‐PPARα‐PGC1α axis, which results in improved lipid metabolism and mitochondrial function and biogenesis. Pharmacological activation of these receptors using adiponectin receptor agonists such as AdipoRon has been explored for their potential to reverse ceramide‐induced lipotoxicity and improve cardiometabolic health [159].

In addition to their role in T2D, ceramides have been implicated as biomarkers in cardiovascular disease (CVD), specifically as biomarkers of cardiovascular risk. Dysregulation of ceramide and sphingolipid metabolism has been reported as a contributing factor in cardiac lipotoxicity and hypertrophy [160]. In plasma, ceramides are transported primarily by lipoproteins, with approximately 60% bound to low‐density lipoprotein (LDL) and 24% associated with high‐density lipoprotein (HDL). They are also enriched in extracellular vesicles, including exosomes and lipid vesicles secreted by various cell types. Ceramide‐based risk scores, including species such as Cer(d18:1/16:0), Cer(d18:1/18:0), and Cer(d18:1/24:1), are recognized as stronger predictors for the assessment of CVD risk compared to traditional biomarkers such as LDL cholesterol and high‐sensitivity CRP [161]. Taken together, these scores enable more precise cardiovascular risk stratification and specifically could improve the identification of high‐risk patients [162]. These lipids accelerate the uptake and aggregation of LDL particles and their infiltration into the arterial wall, potentiating endothelial dysfunction and driving apoptotic and inflammatory responses within vascular tissues [163]. Plasma ceramide concentrations are elevated in patients with obesity and T2D, and have emerged as early predictive biomarkers of T2D onset [164]. Notably, increased levels of dihydroceramides, precursors in the ceramide biosynthesis pathway, have been detected years before clinical diagnosis of T2D, highlighting their prognostic potential. Specific ceramide species, such as Cer(d18:1/18:0), Cer(d18:1/20:0), and Cer(d18:1/24:1), are significantly elevated in T2D patients compared to healthy individuals, and are inversely correlated with insulin sensitivity. This suggests a causal role in the development of IR [165].

While statins are widely prescribed for lowering LDL cholesterol by inhibiting HMG‐CoA reductase, the rate‐limiting enzyme in the mevalonate pathway of cholesterol synthesis, this reduces the synthesis of downstream isoprenoid intermediates such as farnesyl and geranylgeranyl pyrophosphates. This adversely impairs insulin signaling, GLUT 4 translocation, and mitochondrial function, thereby diminishing glucose uptake into cells and promoting IR. This disruption increases the risk of new‐onset or incident T2D, particularly at higher doses of statin or in individuals with pre‐existing metabolic risk. Moreover, while statins and proprotein convertase subtilisin/kexin‐type 9 (PCSK‐9) inhibitors (traditionally used for cholesterol reduction) may modestly reduce circulating plasma ceramide levels via reducing lipid intermediates, they do not directly target ceramide biosynthesis or ceramide‐induced inflammation and β‐cell function. This highlights the need for more targeted interventions that directly address ceramide‐driven metabolic dysfunction in T2D and cardiometabolic disease [166, 167].

In contrast, ceramide inhibitors directly block enzymes in the ceramide synthesis pathway (e.g., serine palmitoyl transferase (SPT) or ceramide synthases), reducing toxic ceramide accumulation associated with IR and inflammation. Preclinical studies of compounds such as myriocin or fumonisin B1 have demonstrated targeted and potent reductions in circulating and tissue ceramide concentrations, with corresponding improvements in glycemic control, adipose tissue inflammation, and β‐cell preservation. Combining ceramide inhibitors with statins may offer synergistic benefits by simultaneously targeting atherogenic lipids and lipotoxic sphingolipids, preserving the cardiovascular benefits of statins while counteracting their diabetogenic effects. This dual approach holds promise for patients at the intersection of dyslipidemia, IR, and cardiovascular disease, providing a more precise pathway‐specific model of cardiometabolic therapy [168].

Glucagon‐like peptide‐1 receptor agonists (GLP‐1 RA), GLP‐1 analogues, are a class of therapeutic agents used in the management of T2D that have also demonstrated cardioprotective effects in part by mitigating ceramide accumulation. The GLP‐1 RA liraglutide in particular has been shown to reduce hepatic levels of key ceramide species (notably C16:0 and C24:0), decreasing liver inflammation, enhancing insulin secretion, reducing β‐cell apoptosis, and promoting weight loss and improved lipid metabolism. When co‐prescribed with statins, GLP‐1 RAs may help offset statin‐induced diabetogenic effects, offering a synergistic strategy for both glycemic and cardiovascular risk management. According to the 2023 American Diabetes Association guidelines, the use of GLP‐1 RAs has emerged as a novel and effective therapeutic approach, especially in individuals with established atherosclerotic cardiovascular disease. Simultaneous integration of ceramide profiling alongside routine lipid biomarkers may improve the diagnostic accuracy of metabolic diseases like T2D. Measuring circulating ceramides is a potentially promising diagnostic tool for monitoring disease progression and predicting cardiovascular events more effectively than traditional lipid analysis alone.

## Oxidative stress

In T2D, mitochondrial dysfunction disrupts ROS production and clearance due to impaired ETC activity, uncoupling protein (UCP) dysregulation, and electron leakage during ATP synthesis. Overnutrition, sedentary lifestyles, and oxidative stress amplify ROS production by 5–10% [95, 169]. Additional sources include xanthine oxidase, ER stress, NADPH oxidase (NOX), NOS, and myeloperoxidase, with mitochondrial superoxide generation and NOX being the major contributors to T2D and vascular complications [170, 171, 172]. Redox imbalance in T2D, involving nine key genes, drives IR, inflammation, and hypertension through pathways such as renin‐angiotensin system upregulation, endothelial dysfunction, lipid peroxidation, β‐cell damage, and advanced glycation end product (AGE) formation (e.g., HbA1c). Hyperglycemia exacerbates ROS production through PKC activation, hexosamine and polyol pathway flux, and enhanced glycation processes [21, 170].

Reduced glutathione (GSH) is critical for antioxidant defense, ROS detoxification, protein folding, DNA repair, and regulation of cell proliferation and apoptosis. GSH also serves as a cysteine depot and supports antioxidant vitamins C and E [173, 174]. β‐cells with low antioxidant enzyme levels are particularly vulnerable to oxidative stress under glucolipotoxic conditions, leading to impaired redox signaling, β‐cell dysfunction, and IR [173, 175, 176, 177]. Combined ER and oxidative stress in T2D arise from increased metabolic demand, misfolded protein accumulation, and chronic inflammation [178]. Pro‐inflammatory pathways including JNK and MAPK accelerate β‐cell death, thereby reducing β‐cell mass [179] (Fig. 2). Oxidative stress also drives the progression of diabetic complications, such as CVD, non‐alcoholic fatty liver disease (NAFLD), and diabetic retinopathy [180].

## The way forward: Subtyping T2D

### Beyond multi‐omics research: Integrative technologies

Pathway analysis tools (computational methods and software that map large‐scale biological data onto known pathways) can interpret multi‐omics results and highlight coordinated alterations in T2D. By overlapping genomic, transcriptomic, and metabolomic data with established pathway databases (KEGG, Reactome, Gene Ontology), researchers can generate hypotheses on mechanisms underlying T2D and potentially identify novel biomarkers for early detection or therapeutic interventions. Efforts to dissect T2D heterogeneity using data‐driven clustering began with Ahlqvist *et al*. (2018), who performed unsupervised hierarchical cluster analysis on clinical data from over 9000 newly diagnosed Swedish diabetics [181]. Five distinct T2D clusters were identified: severe autoimmune diabetes (SAID), severe insulin‐dependent diabetes (SIDD), severe insulin‐resistant diabetes (SIRD), mild obesity‐related diabetes (MORD), and mild age‐related diabetes (MARD), each exhibiting unique characteristics related to diabetic complications and underlying pathophysiology [182]. Two subtypes (SAID and SIDD) were characterized by impaired β‐cell function, while the remaining three reflected different forms of IR: one obesity‐mediated, one by abnormal lipodystrophy‐like fat distribution, and one by altered hepatic lipid metabolism. These findings highlighted that distinct biochemical pathways drive disease progression in different patient subgroups.

Subsequent genetic analyses, such as Udler *et al*.'s genetic clustering analysis, further emphasized T2D's polygenic architecture. By integrating 94 independent T2D genetic variants with 47 diabetes‐related traits from GWAS of numerous T2D cohorts, clusters revealed differential enrichment for tissue‐specific genetic regulators of gene expression, including enhancers and promotors in distinct metabolic pathways [183]. This approach linked genetic risk factors to metabolic pathways, enhancing our mechanistic understanding of T2D subtypes.

Further refinement came from high‐throughput analyses of GWAS data, which identified ten clusters linked to disruptions in core metabolic pathways, such as β‐cell function, lipoprotein metabolism, and sex hormone‐binding globulin (SHBG) [184]. These clusters revealed tissue‐specific epigenomic enrichment, and many overlapped with comorbidities like coronary artery disease and chronic kidney disease [185]. Moreover, cluster‐specific polygenic risk scores (PRS) were grouped with clinical presentation across GWAS and replicated in an independent biobank cohort [186]. However, although all clusters carried T2D risk alleles, they differed in their metabolic outcomes, highlighting the interplay of genetic and biochemical pathways in shaping heterogenous T2D phenotypes [186].

Although these studies advanced our understanding of subtype‐specific pathways, most to date have relied on static‐based cross‐sectional data. However, T2D is inherently dynamic, marked by temporal fluctuations in β‐cell function, insulin sensitivity, and inflammatory burden. This highlights the importance of interorgan cross talk on the bidirectional communication among metabolically active organs such as the liver, skeletal muscle, adipose tissue, the gut, and pancreas, which work in concert to regulate glucose and lipid homeostasis.

As new T2D‐associated loci are discovered and additional GWAS trait summary statistics become available, more finely resolved pathway‐informed subtypes are likely to emerge, improving our understanding of disease pathogenesis and supporting the development of subtype‐specific therapeutic strategies. Nonetheless, translating genetic and molecular insights into clinical subtypes requires a systems‐level approach that accounts for the dynamic and interconnected nature of disease progression.

### Overlapping signaling nodes in T2D: Interorgan cross talk during disease development and progression

T2D pathogenesis as a systemic metabolic disorder extends beyond isolated cellular defects to encompass a breakdown in interorgan communication [187]. In T2D, this communication becomes dysregulated, resulting in widespread metabolic imbalance and chronic inflammation. Understanding and targeting these overlapping signaling nodes and dysfunctional communication networks is essential for more effective prevention, earlier detection, and tailored interventions for T2D. Under physiological conditions, organs communicate to adjust substrate fluxes and maintain energy balance. Pancreatic insulin and glucagon regulate hepatic glucose output; skeletal muscle‐derived myokines influence hepatic gluconeogenesis and adipose lipolysis; adipokines such as leptin and adiponectin modulate insulin sensitivity and appetite; and the gut–brain axis relays nutrient and satiety signals via incretins and autonomic pathways. Skeletal muscle, long known for glucose disposal, is now recognized to function as an endocrine organ secreting myokines such as IL‐6 and meteorin‐like (METRNL), which enhance thermogenesis and lipid oxidation [188]. During exercise, these myokines promote metabolic health, but in sedentary or obese individuals, their altered profiles contribute to systemic inflammation and IR [189].

Dysregulation of these feedback systems initiates a cascade of maladaptive signaling. In early IR, visceral adipose tissue expands and becomes infiltrated by macrophages, triggering inflammation and the release of excess FFAs. These accumulate ectopically in the liver and muscle, where they impair insulin signaling through lipotoxic mechanisms. Simultaneously, adiponectin levels decline while pro‐inflammatory cytokines such as TNF‐α and IL‐6 increase, further exacerbating IR (Fig. 1).

Metabolites including diacylglycerols (DAGs), ceramides, and amino acids such as BCAAs serve not only as energy substrates but also as potent signaling molecules. These molecules activate stress kinases such as PKC and JNK, thereby inhibiting insulin signaling in a tissue‐specific yet system‐wide manner. The pancreas is also affected by this cross‐organ dysfunction: chronic exposure to palmitate, inflammatory cytokines, and organokines contributes to β‐cell stress, mitochondrial dysfunction, and impaired insulin secretion. Emerging data implicate exosomes and microRNAs derived from liver and adipose tissue as regulators of gene expression in pancreatic islets, providing additional mechanistic links between peripheral metabolic disturbances and endocrine failure.

One of the most critical disruptions in T2D occurs within the pancreas–liver axis. In T2D, hepatic IR allows continued glucose output despite hyperglycemia. At the same time, inadequate insulin secretion and inappropriate glucagon release from α‐cells amplify hepatic gluconeogenesis. This results in a chronic oversupply of glucose, driving prediabetic hyperglycemia and accelerating progression to overt T2D (Fig. 1).

Understanding interorgan cross talk remains methodologically challenging. These signaling networks are inherently dynamic and involve feedback loops that fluctuate with nutritional status, circadian rhythms, and genetic factors. For example, IL‐6 acts as an insulin sensitizing myokine during intense exercise but becomes pro‐inflammatory in chronic obesity [189]. Similarly, ceramides and adipokines can exert either protective or pathogenic effects depending on their concentration, exposure, and tissue, complicating mechanistic interpretation and therapeutic targeting. Capturing such temporal resolution is critical, emphasizing the need for longitudinal multimodal datasets and computational tools with high temporal resolution to identify the shifting physiological landscape of T2D and identify windows for intervention.

Machine learning (ML), computational algorithms that learn patterns from data—supervised models, for example, Random Forest (RF), Support Vector Machine (SVM), and XG Boost, can be used to predict known outcomes. Unsupervised models, such as clustering, can uncover hidden groupings in the absence of pre‐labeled outputs. Multi‐omics integration, combining transcriptomic, metabolomic, lipidomic, and proteomic data paired with ML approaches, can uncover hidden patterns of disease heterogeneity. ML algorithms capable of modeling nonlinear interactions and incorporating longitudinal data can identify distinct T2D subtypes (or endotypes) based on interorgan signaling profiles. Unsupervised clustering of omics datasets has identified immune‐dominant, hepato‐centric, and adipose‐driven phenotypes based on lipidomics, inflammatory cytokines, and metabolomic signatures (e.g., BCAA, ceramides). For example, unsupervised analysis of skeletal muscle transcriptomics from T2D patients has been clustered into subtypes with elevated myokine expression influencing hepatic gluconeogenesis and altered AMPK/mTOR signaling pathways that regulate cross‐tissue glucose uptake and usage.

Temporal modeling, using longitudinal datasets to map disease trajectories, can be used to identify T2D patients who transition between subtypes over time. For instance, progression from MOD to SIDD can be detected by declining C peptide levels and impaired insulin secretion marking progressive β‐cell failure. Deep learning tools, an advanced subset of ML capable of modeling high‐dimensional, nonlinear data, are increasingly being used to integrate multi‐omics and clinical data to model such cross talk and feedback between tissues. Such integrative approaches, known as “precision diabetology,” have the potential to revolutionize diagnosis and treatment by enabling personalized, system‐oriented therapeutic strategies. Ultimately, understanding and targeting these interorgan signaling nodes from lipotoxicity and inflammation to gut–brain–adipose axes is essential for more effective prevention, earlier diagnosis, and more effective intervention strategies in T2D.

### Pathways driving disease mechanisms and therapeutic targeting

**Fig. 3:**
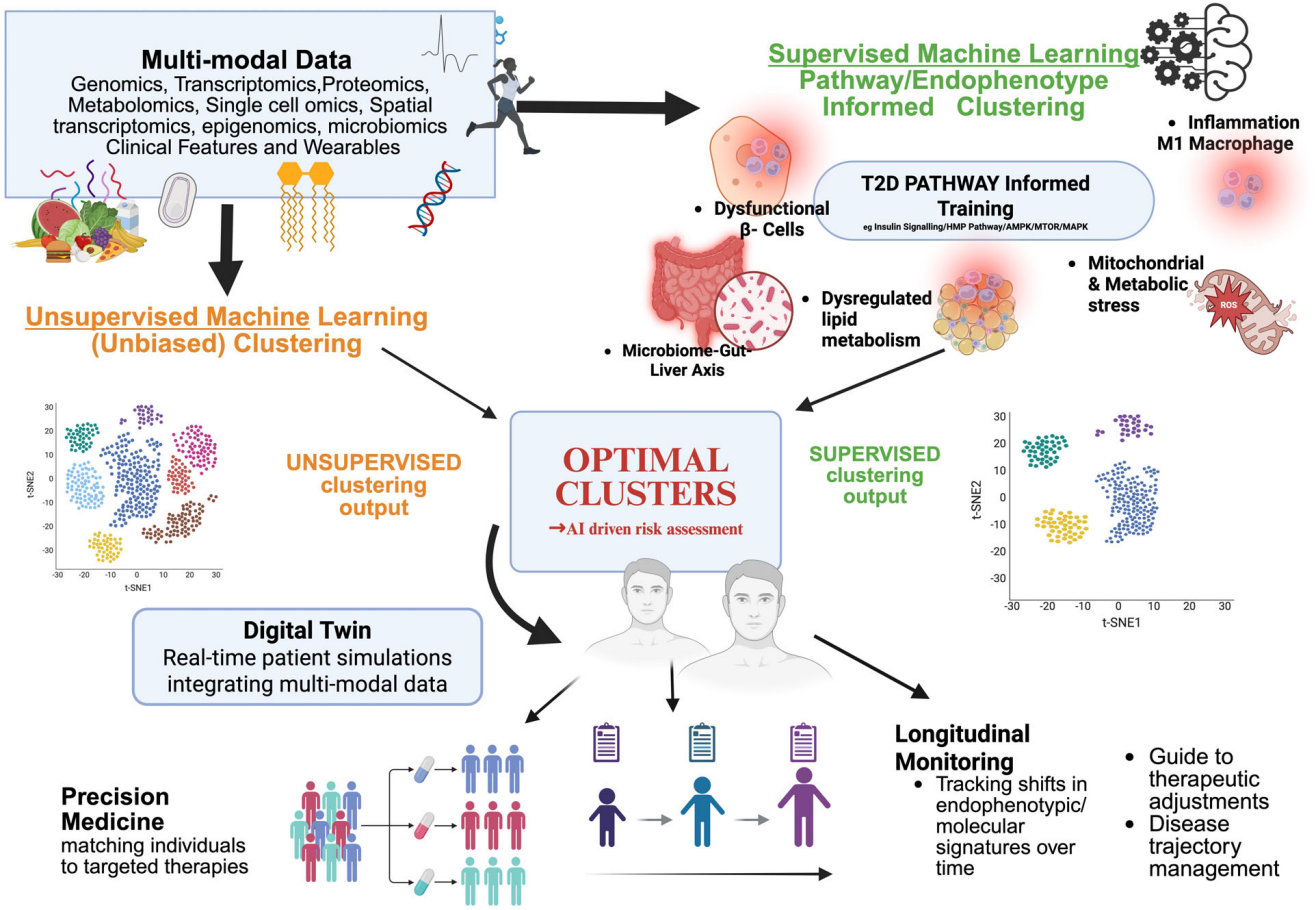
Pathway‐driven framework for defining T2D endophenotypes and their integration into AI‐assisted precision medicine. T2D heterogeneity is stratified into molecular‐based distinct clusters driven by dysregulated pathways, which include inflammation, lipid metabolism, mitochondrial dysfunction, and altered glucose homeostasis. The inflammatory endophenotype, characterized by M1 macrophage activation and elevated TNF‐α, IL6, IFNγ, and CRP, contributes to pancreatic β‐cell dysfunction, insulin resistance, and systemic metabolic stress. Gut dysbiosis and endotoxemia further exacerbate inflammatory signaling, creating a feedforward loop of metabolic dysregulation. Aberrant lipid metabolism, leptin resistance, and adiponectin suppression promote insulin resistance and energy imbalance, while mitochondrial inefficiency leads to increased gluconeogenesis, glycogen storage, and ketogenesis. Metabolic stress is compounded by hyperactivation of glycolysis, the TCA cycle, and the ETC, alongside dysregulated MTOR, AMPK, and MAPK pathways, driving oxidative stress and apoptosis of pancreatic islet cells. Multi‐omics integration (genomics, transcriptomics, proteomics, metabolomics, epigenomics, microbiomics) enables deep molecular characterization of these subtypes. Single‐cell sequencing, including spatial transcriptomics, further refines disease stratification by capturing cellular heterogeneity within pancreatic islets. AI‐assisted precision medicine exploits machine learning to identify disease biomarkers, define phenotypic spectra, and predict individualized therapeutic responses based on pathway dysfunctions. This transforms T2D disease management and advances personalized intervention strategies by aligning molecular endophenotypes with targeted treatments.

Intermediate phenotypes such as BMI, fasting insulin, and lipid levels serve as markers that reflect disruptions in specific metabolic and signaling pathways in T2D [182, 190]. For example, pro‐inflammatory pathways (NF‐κB, JNK) and cytokine signaling (e.g., CRP, IL‐1β) drive IR and β‐cell stress, while PPARγ and leptin signaling mediate obesity‐driven IR. Adopting a pathway‐centric perspective enables a more precise classification of T2D subtypes and supports the development of targeted therapeutic strategies that address the underlying mechanisms of disease progression (Fig. 3). An illustration is that of mild obesity‐related diabetes (MORD): here, PPARγ variants lead to increased adipogenesis and obesity, contributing to IR. Such patients might benefit from therapies that activate PPARγ or reduce adipose inflammation. This pathway‐centric layered understanding aligns with the “palette model” of T2D risk factors, in which each pathway or risk factor is represented as a color on an artist's palette. Individuals within a specific T2D subgroup share a distinct combination of “colors,” while overlapping traits among subgroups reflect blended colors. This framework emphasizes the interconnected and complex nature of T2D pathogenesis, highlighting a continuum of shared and unique risk factors that drive the disease's heterogeneity [191].

Distinguishing between overlapping and pathway‐specific therapeutic targets presents a challenge in the management of T2D. Core pathological features–IR, β‐cell dysfunction, and chronic inflammation converge on shared intracellular signaling nodes or pathways (e.g., mTOR, JNK, and NF‐κB). These nodes serve as central regulators of both metabolic and immune processes, and their ubiquitous activation across tissues complicates targeted intervention. Indiscriminate inhibition risks widespread unintended systemic effects, emphasizing the need for pathway‐specific strategies tailored to the disease stage, the tissue type, and the patient phenotype. For example, broad inhibition of mTOR using agents like rapamycin suppresses both mTORC1 and mTORC2. While hyperactivation of mTORC1 is associated with IR, mTORC2 is essential for Akt‐mediated insulin signaling and β‐cell survival. Inhibition of mTORC2 may therefore worsen glycemic control and compromise β‐cell function. Similarly, systemic inhibition of JNK, although capable of improving insulin sensitivity by reducing IRS1 phosphorylation, can impair β‐cell survival under oxidative stress and adversely affect immune cell function, particularly in macrophages and T lymphocytes. NF‐κB, a master regulator of cytokine production and immune homeostasis, also plays an essential role in maintaining gut epithelial barrier integrity. It controls the expression of tight junction proteins (e.g., occludin, claudin), mucins, and antimicrobial peptides, as well as coordinating epithelial repair processes. Broad inhibition of NF‐κB disrupts these protective functions, resulting in defective tight junction assembly, increased epithelial permeability (“leaky gut”), impaired mucosal defense, and delayed epithelial restitution. Paradoxically, therefore, indiscriminate suppression of NF‐κB may undermine mucosal immunity and amplify systemic immune activation, despite its anti‐inflammatory intention. Similarly, overactivation of AMPK, though generally beneficial for metabolic regulation, can have adverse outcomes such as hypoglycemia and muscle catabolism under certain conditions. Such examples emphasize the risks associated with nonspecific pathway targeting and reinforce the need for precision therapeutic strategies that regulate signaling pathways in a manner that is biologically function‐specific, organ‐specific, spatially relevant, and aligned with disease progression.

Emerging tools in single‐cell and spatial transcriptomics offer high‐resolution insight into temporal and spatial patterns of signaling pathway activation. These tools facilitate the distinction between adaptive and pathological responses, improving target selection. For instance, selective inhibition of S6 kinase 1 (S6K1), a downstream effector of mTORC1, can attenuate IR while preserving mTORC2 activity, which is critical for maintaining β‐cell proliferation and viability [192, 193]. Integrative multi‐omics approaches such as the combination of lipidomic and proteomic datasets enable the identification of upstream drivers of the mTOR–JNK axis, distinguishing between lipid‐mediated stress (e.g., ceramide accumulation) and cytokine‐induced inflammation [194]. Biomarker profiling can further refine treatment stratification; elevations in IL‐1β or TNF‐α suggest a dominant immune‐mediated pathology, whereas increased circulating DAGs or ceramides point toward dominant metabolic dysregulation.

Given the multifactorial nature of T2D pathogenesis, therapeutic strategies must be aligned with the underlying mechanisms in a patient‐specific manner (Table 2). Insulin sensitizers, particularly metformin and pioglitazone, are most effective in individuals with obesity‐driven IR, hepatic steatosis, or lipotoxicity [195]. Metformin activates AMPK, thereby suppressing hepatic gluconeogenesis, inhibiting lipogenesis, and reducing inflammatory signaling through mTORC1 and NF‐κB pathways [196]. Pioglitazone, a PPARγ agonist, is particularly beneficial in patients with visceral adiposity, elevated ceramide levels, or reduced adiponectin. In addition to improving insulin sensitivity, it promotes macrophage polarization from pro‐inflammatory M1 to anti‐inflammatory M2 phenotypes and reduces adipose tissue fibrosis [197].

**Table 2:** Mechanism of action of anti‐diabetic drug classes and their axis‐specific effects.

Drug class	Example drug	Molecular target/pathway	Axis affected	T2D subtype	Mechanism summary
Biguanides	Metformin	Activates AMPK → ↓ hepatic gluconeogenesis	Metabolic	MOD, MARD	Improves hepatic insulin sensitivity and reduces hepatic glucose production via AMPK activation
TZD	Pioglitazone	Activates PPAR‐γ → ↑ adiponectin, ↓ inflammatory cytokines	Metabolic + Inflammatory	MOD, SIRD	Improves insulin sensitivity in adipose and muscle; anti‐inflammatory effects via PPAR‐γ activation
Sulphonylureas	Gliclazide	Stimulates insulin secretion via ATP‐sensitive K^+^ channel inhibition	Metabolic	SIDD, MARD (early)	Enhances insulin secretion from pancreatic β‐cells
DPP4 inhibitors	Sitagliptin	Inhibits DPP‐4 enzyme → prolongs GLP‐1 action	Metabolic	MOD, MARD	Increases incretin activity, enhancing insulin secretion and suppressing glucagon
GLP‐1 receptor agonists	Liraglutide	Activates GLP‐1 receptor → ↑ insulin, ↓ glucagon	Metabolic + Inflammatory	SIDD, MARD, MOD	Improves glycemic control, delays gastric emptying, reduces weight and inflammation
SGLT2 inhibitors	Empagliflozin	Inhibits SGLT2 in proximal tubule → ↑ urinary glucose excretion	Metabolic + Cardiovascular	SIRD, MARD	Lowers blood glucose by promoting glucosuria, offers cardiorenal protection

In contrast, GLP‐1 RAs, such as liraglutide and semaglutide, are best suited to patients with early β‐cell dysfunction, central obesity, overfeeding‐driven hyperinsulinemia, or increased cardiovascular risk. These agents enhance glucose‐stimulated insulin secretion, inhibit glucagon release, slow gastric emptying, and promote satiety, contributing to weight reduction and improved glycemic control. Their anti‐inflammatory properties, favorable effects on hepatic steatosis, and demonstrated cardiovascular benefit in large outcome trials make them a rational choice in individuals with mixed metabolic‐inflammatory profiles, including those with metabolic‐associated steatotic liver disease (MASLD) [198, 199, 200].

In patients with a dominant immune‐inflammatory phenotype, characterized by elevated IL‐6, CRP, or histological evidence of macrophage infiltration in adipose tissue, immune‐targeting agents may offer therapeutic benefit. IL‐1β antagonists (e.g., anakinra) and TNF‐α inhibitors can attenuate inflammation at the source and mitigate cytokine‐induced IR [201]. Pioglitazone, although traditionally classified as an insulin sensitizer, may also serve in this context due to its immunomodulatory effects [195].

The convergence of metabolic and immune dysfunction in T2D highlights the need to shift from uniform glycemic targets toward mechanistic and pathway‐specific treatment strategies. Overlapping disruptions in both metabolic and immune‐inflammatory pathways make therapeutic stratification challenging. For example, the decision to prioritize targeting metabolic axis signaling (e.g., AMPK‐mTOR) or the immune‐metabolic axis (e.g., macrophage polarization) depends on the dominant pathogenic driver in a given individual and the stage of disease progression. Activation of AMPK with agents like metformin offers several benefits including the restoration of cellular energy homeostasis, a reduction of inflammation, improved insulin sensitivity, and enhancement of autophagy at the cellular level, which helps mitigate organelle stress caused by lipotoxicity. This pathway is particularly effective in insulin‐resistant, obese subtypes characterized by mitochondrial dysfunction, ER stress, and lipid overload. Conversely, in lean individuals with T2D, systemic inflammation often precedes IR. In such cases, therapeutic targeting of macrophage polarization from pro‐inflammatory M1 to anti‐inflammatory M2 phenotypes is more relevant. Therapies such as PPARγ agonists (e.g., pioglitazone), TNF‐α inhibitors, and IL‐1β antagonists (e.g., anakinra) can reduce inflammation by promoting M2 macrophage polarization and insulin sensitivity. Targeting the immune‐metabolic axis has been shown to be effective in inflammatory T2D subtypes or in late‐stage disease where β‐cell failure is primarily driven by cytokines. Several studies have shown that elevated IL‐6 and TNF‐α predict β‐cell decline more strongly than IR markers such as homeostatic model assessment of insulin resistance (HOMA‐IR), indicating the need for immune‐modulatory interventions [202, 203, 204].

In patients with overlapping phenotypes, longitudinal phenotyping becomes essential. By integrating temporal multi‐omics, immune profiling, and biomarkers, digital twin (DT) technology can simulate individual disease trajectories, identify the dominant dysfunctional axis, and forecast therapeutic responses [205]. Such dynamic, AI‐driven models will resolve ambiguity in patient stratification and support adaptive, personalized interventions that evolve with disease progression [206].

A rapidly emerging layer in this framework is the gut microbiome. Gut microbiomics, the systems‐level study of microbial communities in the gut and their functional interactions with the host, utilizes high‐throughput technologies such as 16SrRNA sequencing, shotgun metagenomics, metatranscriptomics and metabolomics to map complex host–microbiome relationships [207]. Precision nutrition, an application of microbiomics, aligns closely with the pathway‐centric paradigm of T2D subtyping. Personalized dietary interventions informed by an individual's genetic profile, microbiome composition, and metabolic profile can regulate key molecular pathways implicated in T2D [208]. For example, dietary polyphenols have been shown to downregulate pro‐inflammatory mediators (NF‐κB, JNK), while fermentable fibers selectively support SCFA‐producing bacteria which enhance glycemic control, insulin sensitivity and lipid metabolism. In obesity‐driven IR, dietary strategies that activate PPARγ or reduce BCAA intake can attenuate mTOR hyperactivation, thereby improving metabolic outcomes. When coupled with metabolic and clinical data, precision nutrition offers a level of pathway‐specificity that not only parallels, but may surpass pharmacological interventions in the targeted management of T2D subtypes [209]. ML has accelerated this process by supporting phenotypic prediction, biomarker discovery, and patient stratification. Supervised ML models such as SVM and RF have demonstrated high predictive accuracy in identifying taxa (e.g., *Bifidobacterium*, *Roseburia inulinivorans*, *Allisonella*, *Prevotella*) associated with T2D risk. Using these features, researchers have constructed a microbiome risk score (MRS) which correlates positively with future increases in blood glucose and is linked to specific gut‐derived metabolites [210]. While ML has shown considerable promise in uncovering complex relationships between host–microbiome and T2D, these approaches face challenges. These include “the curse of dimensionality”, high interindividual variability (e.g., as observed in gut microbiota profiles of monozygotic twins), limited replicability across cohorts and the constraints of stool‐based sampling which complicates feature selection, model generalizability and hinder biological interpretations. Emerging technologies such as spatial metagenomics and single‐cell microbiome profiling are significantly enhancing our ability to interrogate host–microbiome interactions at unprecedented resolution [211]. By integrating gut microbiome GWAS (mGWAS) with single‐cell transcriptomic data, researchers can now dissect microbe–host cross talk with fine‐tuned spatial and cellular specificity, revealing how microbial signals influence distinct host cell populations and disease pathways [212]. These findings represent a critical step forward in decoding the bidirectional cross talk between microbes and host cells and lay the foundation for microbiome‐informed precision medicine strategies [213, 214]. The emerging use of organ‐on‐a‐chip and gut organoid technologies is promising as it enables modeling of host–microbe interactions, thus further deepening our understanding of the gut‐organ axis in health and disease [215].

### Transformative potential of multi‐omics integration

Bioinformatics techniques such as marker set enrichment analysis (MSEA) and weighted key driver analysis (WKDA, e.g., MergeOMICS) have been applied to identify pathways and biological processes enriched in multi‐omics T2D data. These tools have identified critical hub genes and metabolites that act as key regulators of disease in T2D pathways [44, 216]. Single‐cell sequencing has emerged as a transformative technology, allowing researchers to interrogate gene dysregulation and cellular heterogeneity [217]. By examining chromatin accessibility, gene expression, and functional states in individual β‐cells from non‐diabetic, prediabetic, and T2D individuals, single‐cell genomics can link specific genetic variants to disease mechanisms [43, 52]. Single‐cell analyses have identified distinct endophenotypes— measurable biological traits linking genetic predisposition to disease, acting as an intermediate between molecular pathways and clinical phenotypes—across various cell types (β‐cells, adipocytes, skeletal muscle cells, hepatocytes, immune cells), revealing each cell type's contribution to disease progression. The advent of single‐cell multi‐omics enables accurate identification of pancreatic cell types and their transcriptomic changes during islet formation and diabetes onset [218, 219] (Fig. 3).

Advances in multi‐omics integration coupled with ML now allow for the extraction of microbial signatures and metabolic features that correlate with disease trajectories.

Lipidomic profiling and ceramide biology offer another promising avenue for T2D subtyping. By quantifying circulating ceramide species (e.g., Cer 16:0; Cer18:0, Cer24:1) using lipidomics and integrating genomic variants in ceramide synthase genes with transcriptomic and metabolomic data, it is possible to identify ceramide‐driven subtypes of T2D. Unsupervised ML can cluster patients based on ceramide‐driven endotypes. Integration of ceramide profiling into a precision medicine framework has the potential to develop personalized pathway‐specific therapy (e.g., identify patients likely to benefit from targeted agents such as myriocin, fumonisin B1, or AdipoRon). This molecular stratification represents another distinct therapeutic layer operating alongside and in concert with gut‐microbiome‐informed nutrition and traditional glucose‐lowering pharmacotherapies.

### Challenges to subtype classification and the role of digital twins

Longitudinal studies using advanced technologies have enabled the tracking of β‐cell transitions from healthy to diseased states, revealing patterns of metabolic reprogramming and uncovering distinct subtypes of dysfunctional cells [52, 56, 220]. Longitudinal cohort studies, including IMI DIRECT and RHAPSODY, along with emerging DT technologies, offer powerful platforms to simulate disease progression and predict therapeutic response. These tools guide the development of individualized intervention strategies [41, 65, 221]. By integrating clinical, molecular, and multi‐omic data, including genomic, transcriptomic, metabolomic, and proteomic layers, DT models can inform optimal timing and sequencing of therapeutic agents, including insulin sensitizers, GLP‐1 RAs, and immunomodulators, thereby enabling precision medicine approaches that dynamically adapt to the evolving pathophysiology of each patient.

Beyond omics, spatial omics techniques integrate single‐cell data with spatial transcriptomic maps, offering a 3D view of cellular organization in pancreatic islets [219, 222, 223]. For example, multi‐omics analysis of islets obtained from metabolically pancreatectomized living human donors spanning the glycemic continuum (from normoglycemic to overt T2D) demonstrated a progressive remodeling of mature β‐ cells in parallel with rising HbA1c levels [223].

The fusion of omics with nanotechnology has given rise to nano‐omics, which integrates nanoscale materials and devices with genomic, proteomic, metabolomic, and transcriptomic analyses. This approach is unlocking unparalleled opportunities for personalized medicine—including high‐resolution insights into cellular processes, enhanced diagnostic accuracy, targeted drug delivery, real‐time monitoring of treatment responses, and enhanced efficacy and prognostics/predictive modeling [224]. The convergence of nano‐omics and personalized medicine represents a transformative leap toward a future where healthcare is tailored to the unique molecular signature of each patient and can adapt as the disease evolves (Fig. 3).

### Overcoming computational challenges for personalized care

The explosion of new technologies in diabetes research and care (from multi‐omics to continuous glucose monitoring devices) has opened doors for more effective disease management. This is essential given T2D's complexity and the necessity for continuous monitoring and adjustment of treatment plans. In this domain, AI is revolutionizing diabetes care by predicting risks and customizing treatment plans to enhance patient outcomes. AI algorithms can analyze large datasets to predict an individual's diabetes risk, interpret results accurately, and customize treatment plans, enhancing patient care and shifting care toward a more proactive and personalized model. The complexity and dimensionality of multi‐omics data necessitate sophisticated bioinformatics and AI platforms and personalized statistical tools for real‐time data integration and visualization [225]. Continuous glucose monitors (CGMs) provide real‐time data on a patient's metabolic processes, allowing healthcare providers to fine‐tune treatment in real time [225, 226, 227]. ML models have successfully clustered T2D patients into precise phenotypic subclasses. By aggregating extensive data on genetic profiles, metabolic markers, and clinical histories, AI methods (such as SVM, genetic algorithms, and deep learning, etc.) can extract relevant features related to endophenotypes, subtypes, and biochemical pathways [228, 229] (Fig. 3). These algorithms then train ML models on large datasets to recognize patterns and predict T2D risk, enabling the deployment of personalized risk scores and tailored intervention strategies. This approach can identify high‐risk individuals *before* clinical symptoms appear, facilitating early intervention and prevention [230].

However, while emerging technologies offer unprecedented insights, they are not sufficient on their own. A deeper focus on the functional pathways driving disease progression is needed to ensure that data‐driven predictions translate into meaningful care. Continually re‐examining the interplay between signaling pathways and systemic metabolic disruptions is vital, prioritizing how molecular and cellular mechanisms interact in each patient, so that interventions target root causes rather than just correlations.

### Digital twins in precision diabetology: A dynamic framework for T2D management

Digital twins (DT) represent a significant advancement in precision diabetology. These dynamic virtual representations of an individual's health trajectory integrate longitudinal, multimodal data, including clinical records, multi‐omics profiles, CGM, behavioral data, and environmental exposures to simulate disease progression and predict individual responses to therapeutic interventions in real time [231, 232]. ML algorithms process this high‐resolution input to enable real‐time phenotyping, and predictive analytics establish personalized feedback loops. Continuously evolving alongside the patient, the DT represents an integrative approach that offers a holistic framework for T2D management: optimizing glycemic control, supporting behavioral change through technological nudges, and potentially reducing the reliance on pharmacological interventions.

Unlike traditional static clustering approaches (e.g., SIDD, SIRD, MOD, MARD) which fail to reflect the dynamic and overlapping nature of T2D subtypes, DTs accommodate temporal changes. Patients often transition between subtypes as their physiology evolves due to changes in environmental exposures, lifestyle, and therapy. Static subtyping can miss such temporal changes that occur with time. DTs resolve this ambiguity by stochastic modeling to simulate disease progress, anticipate subtype transitions, and predict differential treatment responses in real time [205]. Such subtype transitions are clinically meaningful and represent signal changes in underlying biology. Recognizing and preempting these transitions allows clinicians to adapt therapy, such as intensifying treatment prior to irreversible β‐cell failure or introducing inflammatory strategies to curb systemic immune hyperactivation, which can improve outcomes. Moreover, DTs allow for the detection of metabolic rebound events such as rising HbA1c, IR, or surging of inflammatory markers, enabling rapid course correction [205, 233]. By mapping immune‐metabolic axes in real time, DTs shift T2D subtyping from a static classification to a continuously evolving therapeutic decision‐making tool [233] (Fig. 3).

### Temporal pathophysiology and precision medicine in T2D

Different pathogenic pathways dominate at different stages of T2D, a trajectory reflected by the temporal changes in molecular pathology. Early in the disease (prediabetes or new‐onset T2D), hepatic IR and compensatory hyperinsulinemia maintain normoglycemia, prompting β‐cell hyperplasia, an adaptive response to the increased insulin demand. Chronic metabolic stress driven by overnutrition and adiposity induces chronic low‐grade inflammation, macrophage infiltration, and cytokine release, exacerbating IR. Progressive glucotoxicity, lipotoxicity, and inflammation accelerate β‐cell stress and eventual β‐cell dysfunction. Advanced stages are characterized by irreversible β‐cell failure and architectural restructuring of pancreatic islets, including amyloid deposition. Given this temporal and molecular heterogeneity, therapy must be aligned with both the disease stage and the dominant disrupted molecular pathway. In the early stages of T2D, particularly in mildly obese patients with prediabetes or emerging hyperglycemia, the dominant pathophysiological drivers are hepatic IR and excess gluconeogenesis, often accompanied by compensatory hyperinsulinemia. In such cases, metformin is the preferred first‐line therapy. Through activation of AMPK, metformin suppresses hepatic gluconeogenesis, improving hepatic insulin signaling. In contrast, pioglitazone, a PPAR‐γ agonist, targets peripheral IR and inflammation, enhancing glucose uptake via GLUT4 translocation, promoting adipocyte differentiation and adiponectin secretion. It also reduces inflammation (e.g., ↓ TNF‐α, ↑ IL‐10), thereby preserving β‐cell function.

This pathway‐informed approach is the foundation of precision diabetology wherein interventions are mechanistically tailored to an individual's molecular profile and disease trajectory (Fig. 3) [234]. High‐throughput omics technologies (genomics, epigenomics, transcriptomics, metabolomics, proteomics, and lipidomics) have been pivotal in unpacking the complex, stage‐dependent biology of T2D. By profiling metabolic changes across each molecular layer, including single‐cell resolution, researchers can capture molecular snapshots of tissue and cellular dysfunction at the distinct stages of disease pathogenesis and identify stage‐specific biomarkers and therapeutic targets. Repeated multi‐omics measurements (e.g., transcriptomic changes and profiling of metabolites such as ceramides and BCAA) integrated with clinical markers (C peptide, BMI), behavioral inputs (physical activity, sleep patterns), biosensors, and “wearables” (CGM, heart rate variability) enable a comprehensive system‐level model of disease states and transitions. As adaptive models, they capture metabolic and immune axes and model subtype transitions through identification of inflection points, such as rising IL‐6 and CRP with declining C peptide, that signal subtype transitions (e.g., from MOD to SIDD). DTs as adaptive computational models embrace this multimodal data to simulate metabolic and immune axes, providing personalized predictions of disease progression and treatment response in real time.

Recurrent neural networks (RNNs) and other ML models excel at capturing temporal patterns across omics layers [235]. Supervised models can be used to predict disease trajectories and guide personalized interventions, while unsupervised models reveal novel subtypes by identifying unbiased patterns within the data. The ultimate goal is to translate these findings into clinical practice through the development of omics‐based classifiers that extend beyond glycemic indices that dictate treatment selection. Therapies are increasingly designed around disrupted pathways and organ systems rather than symptoms alone. This has resulted in the development of combinational therapies tailored to organ system profiles [236]. Many medications have system‐wide effects. For example, GLP‐1 RAs modulate pancreatic insulin secretion, satiety, and gastric emptying, while SGLT2 inhibitors reduce renal glucose reabsorption and provide cardiorenal benefits [237, 238]. From a clinical perspective, management has moved from the reactive management of established T2D to a proactive, presymptomatic intervention approach. Multisensory wearables such as CGMs that continuously feed data into AI algorithms, allowing for adaptive feedback loops that modify treatment and lifestyle recommendations in real‐time, push the T2D management landscape into a presymptomatic, precision prevention paradigm. AI‐driven algorithms enable early identification and stratification of high‐risk individuals, even at the prediabetic stage, facilitating timely interventions. Preventative pharmacotherapy, such as the administration of GLP‐1 RAs in high‐risk obese individuals, parallels the preventative use of statins in cardiovascular disease, aiming to delay or avert disease onset.

## Future perspectives and conclusion

The study of T2D pathways underpins decades of metabolic research, revealing the disease as a heterogeneous, temporally dynamic condition involving complex interorgan and molecular interactions. Applying multi‐omic technologies to deeply phenotyped epidemiological prospective cohorts allows for a deeper exploration of these intricate disease pathways, uncovering hidden mechanistic insights that surpass the limitations of traditional genetic or clinical analyses. The convergence of bulk‐omics with single‐cell technologies, real‐time biosensor data, and AI‐driven analytics further represents a paradigm shift in T2D research, management, and care. These integrative approaches enable dynamic modeling of disease stages and facilitate the tailoring of therapy based on an individual's evolving molecular profile. Pathway‐informed analyses allow researchers to move from correlation to causation, linking molecular perturbations directly to clinical outcomes, thereby dissecting the biochemical pathways that drive T2D progression and identifying novel therapeutic targets that restore systemic metabolic balance. By integrating multi‐omics insights within clinical workflows, a bidirectional translational framework emerges where clinical phenotypes inform omics hypotheses, and in turn, omics‐guided subtyping informs individualized therapy selection to address the underlying pathophysiology. This dynamic feedback loop facilitates the development of personalized biologically targeted therapies that intervene at the molecular core of the disease. It also holds immense promise for optimizing patient outcomes and shifting the paradigm of T2D care toward prevention, personalization, and system‐level restoration.

As AI and ML models become increasingly embedded into precision medicine workflows, the interpretability of predictive models is essential. Clinicians must be able to trust and implement models that offer transparent, explainable outputs. Tools like ShAP provide model interpretations of how individual variables influence predictions and can be integrated into clinical decision support systems. Knowledge graph models also enhance interpretability as they underpin ML predictions in familiar biology [239]. Equally important is the rigorous validation of AI‐derived recommendations in real‐world settings to ensure reliability, generalizability, efficacy, and ethical integrity. Such models must account for the evolving nature of the metabolic landscape and be continuously retrained as new data emerges from new therapies, lifestyle trends, and evolving phenotypes. In resource‐limited settings where omics profiling may be unaffordable, parsimonious models built on high‐yield clinical variables but informed by omics‐derived research offer a pragmatic path forward.

Finally, as precision platforms become increasingly data‐intensive, patient trust and data governance must be prioritized. The ethical handling of biological, behavioral, and clinical data demands robust systems for privacy, consent, and security. Integration of blockchain infrastructure into healthcare data management presents a promising strategy to ensure data integrity and transparency, building public trust for wide‐scale implementation [240]. The future of T2D management lies in a systems‐level, precision‐guided approach, anchored in biology, empowered by AI, and responsive to real‐time data. DTs, multimodal data integration, and AI‐integrated predictive modeling are poised to transition care from reactive glucose control to proactive, holistic metabolic restoration. This integrative model not only redefines how T2D is diagnosed and treated but also how we understand chronic disease itself, shifting the focus from disease management to molecular‐level prevention.

## Author contributions

SOS and PZ devised the layout of the review, SOS wrote the text, PZ reviewed the several drafts of text, figures, and tables. LQ reviewed the drafts and advised on content. All authors approved the final version of the review.
